# Interleukin 13 promotes long-term recovery after ischemic stroke by inhibiting the activation of STAT3

**DOI:** 10.1186/s12974-022-02471-5

**Published:** 2022-05-16

**Authors:** Di Chen, Jiaying Li, Yichen Huang, Pengju Wei, Wanying Miao, Yaomei Yang, Yanqin Gao

**Affiliations:** State Key Laboratory of Medical Neurobiology, MOE Frontiers Center for Brain Science, and Institutes of Brain Science, Fudan University, 138 Yixueyuan Road, 200032 Shanghai, China

**Keywords:** Interleukin-13, Microglia/macrophage, White matter repair, STAT3, Ischemic stroke

## Abstract

**Background:**

Microglia/macrophages are activated after cerebral ischemic stroke and can contribute to either brain injury or recovery by polarizing microglia/macrophage into distinctive functional phenotypes with pro- or anti-inflammatory properties. Interleukin-13 (IL-13) is an anti-inflammatory cytokine that regulates microglia/macrophage polarization toward an anti-inflammatory phenotype. However, it is not clear whether IL-13 is beneficial after ischemic stroke long-term and the underlying molecular mechanism(s) remain unknown. Thus, we examined the effect of IL-13 on long-term recovery and microglia/macrophage polarization in mice with transient middle cerebral artery occlusion model (tMCAO).

**Methods:**

tMCAO was induced in adult male C57BL/6J mice. IL-13 (60 μg/kg) was administered intranasally starting 2 h after stroke and continued for seven consecutive days. Sensorimotor function, spatial learning and memory function, as well as brain infarct volume were assessed up to 35 days after stroke. White matter integrity was evaluated by electrophysiology, immunofluorescence staining, and transmission electron microscopy. Microglia/macrophage activation was assessed using immunofluorescence staining and quantitative real-time polymerase chain reaction. Changes in immune cells in the brain and the periphery, and expression of IL-13 receptors in different brain cells were detected by flow cytometry. Primary neuron/microglia co-cultures and a STAT3 inhibitor were used for mechanistic studies.

**Results:**

Post-treatment with IL-13 improved long-term neurofunctional recovery and decreased brain tissue atrophy after stroke. Intranasal delivery of IL-13 enhanced the structural and functional integrity of white matter after stroke. Furthermore, the neuroprotection afforded by IL-13 administration was not due to a direct effect on neurons, but by indirectly regulating the anti-inflammatory phenotype of microglia/macrophages. IL-13 treatment also had no effect on peripheral immune cells. Mechanistically, IL-13 improved the long-term outcome after ischemic stroke by promoting the polarization of microglia/macrophages toward the anti-inflammatory phenotype at least partially by inhibiting the phosphorylation of STAT3.

**Conclusions:**

IL-13 promotes white matter repair and improves neurofunctional outcomes after ischemic stroke by modulating microglia/macrophages via inhibition of STAT3 phosphorylation.

**Supplementary Information:**

The online version contains supplementary material available at 10.1186/s12974-022-02471-5.

## Background

Stroke is a major cause of death and disability worldwide, and the global burden of stroke continues to rise as the population ages [1]. At present, recanalization of occluded blood vessels through thrombolysis or thrombectomy with such drugs as tissue plasminogen activator (tPA) is an effective clinical treatment for ischemic stroke approved by the US Food and Drug Administration (FDA) to improve the functional prognosis after acute ischemic stroke [2, 3]. However, tPA has a narrow therapeutic time window and there is a risk of intracerebral hemorrhage, thus most ischemic stroke survivors develop physical disabilities due to the limitations of current therapies.

Neuroinflammation plays a pivotal role in ischemic stroke, mainly manifested in the activation of microglia, which are innate immune cells in the central nervous system (CNS) [4]. Microglia/macrophages are among the first responders to CNS injuries, and they play an important role in neurogenesis, axonal regeneration, synaptic plasticity, white matter integrity, angiogenesis, and vascular repair [5]. As an anti-inflammatory cytokine, interleukin 13 (IL-13) plays a critical role in regulating the inflammatory and immune responses by down-regulating the production of pro-inflammatory factors in microglia [6, 7]. IL-13 is expressed exclusively in activated microglia following injection of lipopolysaccharide (LPS) into the rat cortex where it regulates brain inflammation and contributes to neuronal survival [8]. Moreover, studies have shown that IL-13 mediates the anti-inflammatory phenotype in macrophages [9, 10].

IL-13 is a single-chain glycosylated polypeptide [11] that acts through one of two IL-13 receptors expressed on a variety of cells [12]. The signaling IL-13 receptor, also known as the type II IL-4 receptor, is the heterodimer of the alpha chain of the IL-4 receptor (IL-4Rα, CD124) and IL-13Ralpha1 (IL-13Rα1, CD213a1) [13]. When heterodimerized with IL-4Rα, IL-13Rα1 binds to IL-13 with high affinity and works through the Janus kinase (JAK)–signal transducer and activator of transcription (STAT) pathway [14]. The other receptor that binds with IL-13 is IL-13Ralpha2 (IL-13Rα2, CD213a2), which is thought to act as a decoy receptor with a short intracellular domain [11, 13, 15]. Studies have found that phosphorylated-STAT3 (p-STAT3) is predominantly localized in microglia/macrophages in the post-ischemic brain with increases 6–72 h after ischemic stroke [16]. Inhibiting STAT3 phosphorylation can effectively improve the prognosis of stroke, suggesting that STAT3 activation to an inappropriately high level after focal ischemia might be detrimental [16, 17]. We have previously reported that IL-13 treatment improved neurologic outcomes after traumatic brain injury (TBI) by suppressing pro-inflammatory responses [18]. However, the molecular target of IL-13 in the CNS has not yet been elucidated. Further, the role IL-13 plays in microglia/macrophage-mediated immune responses, white matter injury, and long-term neurological outcomes after ischemic stroke remain unknown.

In the present study, we investigated the role of IL-13 in ischemic stroke and discovered that IL-13 plays a neuroprotective role at least partially by inhibiting the activation of STAT3, altering the polarization of microglia/macrophages, improving brain inflammation, promoting white matter repair, and improving long-term neurofunctional outcomes after ischemic stroke. Therefore, immunomodulation with interleukin-13 is a promising approach to promote long-term functional recovery after stroke.

## Materials and methods

### Data availability

The authors confirm that the data supporting the findings of this study are available within the article and in the Supplementary Data, and can also be obtained from the corresponding author upon reasonable request.

### Experimental animals

Male C57BL/6J mice (8–10 weeks) were purchased from SLAK Laboratory Animal, Shanghai, China. All animals were maintained in a temperature- and humidity-controlled facility with a 12-h light–dark cycle with food and water provided ad libitum. Mice were blindly and randomly divided into experimental or control groups. All animal experiments were approved by the Animal Care and Use Committee of Shanghai Medical College, Fudan University (approval number 20150119-120).

### Ischemic brain injury and intranasal administration of IL-13

Transient middle cerebral artery occlusion (tMCAO) was induced for 60 min in the left brain of adult male C57BL/6 J mice weighing 23–30 g (8–10 weeks) as described previously [19]. Briefly, mice were anesthetized with isoflurane (RWD, Shanghai, China) with the anesthetic plane maintained with 1%-2% isoflurane in a 30% O_2_/70% N_2_ mixture during surgery. During the operation, the rectal temperature was maintained at 37.0 ± 0.5 °C using a temperature-regulated heating pad. Regional cerebral blood flow (rCBF) was measured in all stroke mice using laser Doppler flowmetry (PeriFlux System 5001, Perimed, Jarfalla, Sweden) [20]. Mice that did not display at least 75% reduction of pre-ischemia rCBF levels during tMCAO were excluded from further experimentation. rCBF was imaged using laser speckle (PeriCam PSI System, Perimed, Jarfalla, Sweden). Sham-operated mice underwent the same anesthesia procedure and exposure of arteries without middle cerebral artery occlusion. tMCAO surgeries were performed by an investigator blinded to subsequent animal treatment. Mice were randomly assigned to sham or cerebral ischemia groups and received randomized treatments.

**Fig. 1.**
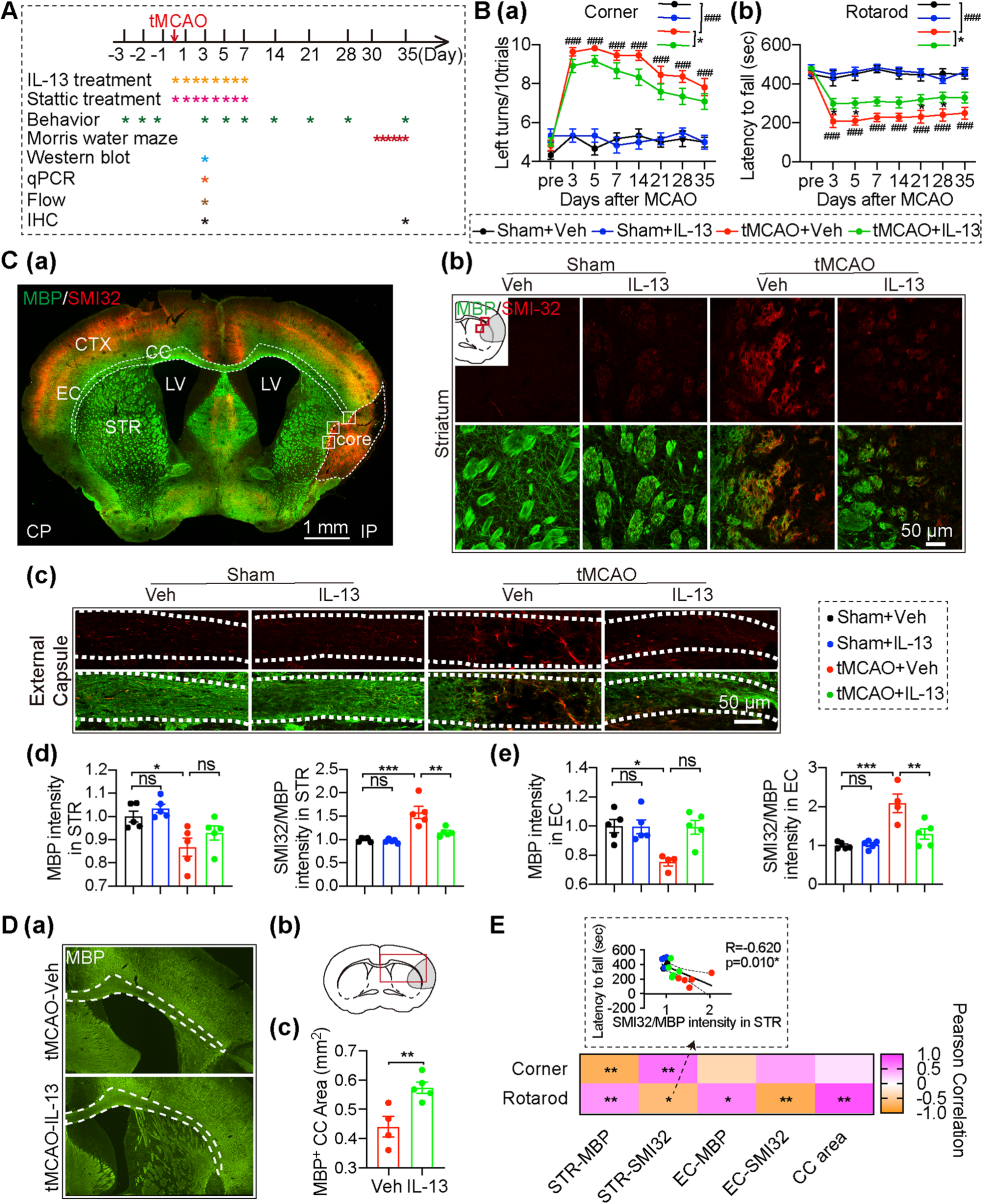
IL-13 post-treatment improves long-term stroke outcomes and enhances structural white matter integrity. **A** Experimental design. **B** Sensorimotor function was assessed by the corner test **Ba** and the rotarod test **Bb** up to 35d after tMCAO or sham operation. *n* = 6 mice for Sham + Veh and Sham + IL-13; *n* = 11 mice for tMCAO + Veh; *n* = 12 mice for tMCAO + IL-13. ^#^tMCAO + Veh vs. Sham + Veh, *tMCAO + Veh vs. tMCAO + IL-13. **C** Double-immunostaining for MBP and SMI32 35d after tMCAO or sham operation. **Ca** illustrates where images in **b**, **c** were taken from. **Cb**, **c** Representative images of MBP (green) and SMI32 (red) immunostaining in the STR and EC. Scale bar: 50 µm. **Cd** Quantification of the MBP fluorescence intensity and the ratio of SMI32 to MBP immunofluorescence intensity in the ipsilateral STR. n = 5/group. **Ce** Quantification of the MBP fluorescence intensity and the ratio of SMI32 to MBP immunofluorescence intensity in ipsilateral EC. n = 4–5/group. **D** The EC areas were measured based on MBP-stained brain slices 35d after tMCAO. **Da** Representative images of MBP (green) immunostaining in the corpus callosum (CC). **Db** illustrates where images in **a** were taken from. **Dc** Quantification of the MBP^+^ area in ipsilateral CC *n* = 4–5/group. **E** Pearson correlation between the animals’ performance and various parameters representing white mater integrity 35 d after tMCAO. Dashed lines: 95% confidence intervals (CIs). Black, blue, red, and green dots represent the Sham + Veh, Sham + IL-13, tMCAO + Veh and tMCAO + IL-13 group, respectively *n* = 3–5/group. All data are presented as the mean ± SEM. *p ≤ 0.05, **p ≤ 0.01, ***p ≤ 0.001 tMCAO + Veh vs. tMCAO + IL-13 or as indicated, # *p* < 0.05, ## *p* < 0.01, ### *p* < 0.001 tMCAO + Veh vs Sham + Veh. One-way ANOVA followed by Bonferroni’s post hoc (**A**, **Cd**, **e**), two-way ANOVA followed by Bonferroni’s post hoc (**B**), unpaired Student’s *t*-test (**Dc**), Pearson correlation (**E**)

Recombinant mouse IL-13 protein (100 μg, 50225-MNAH, Sino Biological Inc, Beijing, China) was dissolved in saline to 150 μg/mL [18]. IL-13 (60 μg/kg body weight) or an equivalent volume of saline was administered intranasally starting two hours after reperfusion followed by intranasal infusion for an additional seven consecutive days (Fig. 1A). Briefly, under isoflurane anesthesia, six drops (no more than 2 μL for each drop) of IL-13 or saline were applied alternately into each nostril with a 2-min interval between drops.

Stattic, a STAT3 inhibitor (CAS 19983-44-9-Calbiochem, Sigma-Aldrich, St. Louis, MO), selectively inhibits activation, dimerization, and nuclear translocation of STAT3. Stattic was dissolved in 5% dimethyl sulfoxide and 95% PBS at a final concentration of 1 mg/mL and stored at − 20 °C. Stattic (4 mg/kg/day) was administered intraperitoneally one hour before tMCAO and then two hours before IL-13 administration and for seven consecutive day thereafter.

### Behavioral tests

All behavioral tests were performed by an investigator blinded to experimental manipulations.

#### Corner test

The corner was made by the intersection of two boards (30 × 20 cm) at a 30-degree angle. During the corner test, mice were placed in the middle of the corner, facing the 30-degree angle, and mice walked freely into the corner. The number of times the mice turned to the left in 10 trails were recorded. In this experiment, only mice with no preference for turning left and right in the pretraining were retained for subsequent experiments. Data measured 1d before tMCAO were recorded as the preoperative data (pre). Repeat testing on the corner test was performed 3d, 5d, 7d, 14d, 21d, 28d, and 35d after tMCAO. Mice with sham operation had no preference for turning to the left and right, while mice with tMCAO turned to the left with a bias.

#### Rotarod test

The Rotarod test was performed with the Rotarod apparatus (Model 47650, Ugo Basile Srl, Varese, Italy). The mice were forced to run on a rotating drum with speeds starting at 5 rpm, accelerating to 40 rpm within 300 s, and maintaining a constant speed of 40 rpm to 500 s. The latency to fall off the rotating rod was recorded. Data were expressed as the mean value from three trials. Mice were pre-trained 1d-3d before tMCAO and data measured 1d before tMCAO were recorded as preoperative data (pre). Repeat testing was performed 3d, 5d, 7d, 14d, 21d, 28d, and 35d after tMCAO.

#### Foot fault test

The mice were placed on a stainless steel frame, which was composed of 1.5 cm × 1.5 cm square grids. The steel frame was 40 cm long, 20 cm wide, and 30 cm above the ground. Mice were pre-trained 1d-3d before tMCAO and data measured 1d before tMCAO were recorded as preoperative data (pre). Repeat testing was performed 3d, 5d, 7d, 14d, 21d, 28d, and 35d after tMCAO. The forelimb foot fault rate was calculated by counting the total number of steps taken by the right forelimb on the grid and the right forelimb missteps (i.e., when the forelimb fell through the grid) during a 1 min videotaped observation period. The hindlimb foot fault rate was calculated by counting the total number of steps taken by the right hindlimb on the grid and the right hindlimb missteps (i.e., when the right hindlimb fell through the grid) from a 1 min videotaped observation period. The foot fault rate was expressed as a percentage of the total number of steps.

#### Adhesive removal test

The adhesive removal test was carried out with 2 × 3 mm tape. Adhesive tapes were applied to the ipsilateral or contralateral forepaw of the mouse to evaluate the sensory and motor function of mice after tMCAO. The amount of time for the mouse to touch and remove the tape was measured up to 120 s, at which point the timer was stopped. Data were expressed as the mean value from three trials. Data measured 1d before tMCAO were recorded as the preoperative data (pre). Repeat testing on the adhesive removal test was performed 3d, 5d, 7d, 14d, 21d, 28d, and 35d after tMCAO.

#### Morris water maze test

Cognitive function was measured using the Morris water maze test (MWM) 30-35d after tMCAO as described previously [21]. In the learning phase, mice were trained on four trials (at four fixed locations) every day between 30d–34d after tMCAO. In each trial the time spent to reach the platform was recorded (within 60 s). If the mouse did not find the platform within 60 s the experimenter guided the mouse to find the platform and the time was recorded as 60 s. At the end of the trials, each mouse was allowed to stay on the platform for 20 s to allow the mouse to memorize the spatial position of the platform. The memory test was performed 35d after tMCAO. The platform was removed and a 60-s probing test was performed on each mouse. The swimming speed and time spent in the goal quadrant were also recorded.

### Measurement of tissue loss

Mice were euthanized and then transcardially perfused with phosphate-buffered saline (PBS, pH 7.4) followed by 4% paraformaldehyde (w/v, dissolved in PBS). Brains were harvested, post-fixed in 4% paraformaldehyde overnight at 4 °C, and then cryoprotected in 30% sucrose in PBS for 2 days at 4 °C. For measurement of cerebral tissue loss, 25-μm-thick frozen serial coronal slices from the brains were sliced on a freezing microtome (Leica). Ten equally spaced coronal brain sections from bregma 1.1 to -1.94 were immunohistochemically stained with rabbit anti-NeuN antibody (1:1000, ab177487, Abcam, Cambridge, MA, USA), a neuronal marker. Cerebral tissue loss was measured on each section with ImageJ image analysis software (National Institutes of health, Bethesda, MD, USA) and the volume of tissue loss was calculated using a numerical integration of the value by subtracting the non-infarcted volume of the ipsilateral hemisphere from the volume of the contralateral hemisphere. Investigators blind to group assignments conducted the quantifications of the volume of cerebral tissue loss. Data are presented as the volume of tissue loss as a percentage of contralateral hemisphere volume.

### ELISA

For assessment of brain IL-13 levels, mice were randomly assigned to the sham or tMCAO group and received IL-13 or saline randomly. IL-13 (60 μg/kg body weight) or an equivalent volume of saline was administered intranasally starting 2 h after reperfusion and then for three consecutive days. Mice were euthanized and perfused with cold PBS then the ipsilateral hemisphere was harvested 3 h after IL-13 or saline administration. Brain IL-13 levels were measured using an ELISA (DY413-05, R&D Systems, Minneapolis, MN, USA) according to the manufacturer’s instructions.

### Western blot

Western blot analysis was performed to assess expression levels of proteins of interest by standard SDS–polyacrylamide gel electrophoresis (PAGE). Briefly, brain tissue was homogenized in lysis buffer (9803S, Cell signaling Technology, Danvers, MA, USA) containing 100 mM phenylmethyl sulfonyl fluoride (1:100), a protease inhibitor cocktail (1:50, 05892970001, Roche, Mannheim, Germany), and a phosphatase inhibitor cocktail (1:10, 04906837001, Roche, Mannheim, Germany). Protein concentrations were measured with a protein detection kit (20201ES, Yeasen, Shanghai, China). The primary antibodies used in this study included: rabbit anti-p-STAT3 (1:500, 9145, Cell signaling Technology, Danvers, MA, USA), mouse anti-STAT3 (1:1000, 9139, Cell signaling Technology, Danvers, MA, USA), rabbit anti-IL-13R Alpha1 (IL-13Rα1, 1:500, LS-C117959, LSBio, Seattle, WA, USA), and mouse anti-β-actin (1:10000, 4967, Cell signaling Technology, Danvers, MA, USA). The secondary antibodies used in this study included: HRP-conjugated goat anti-mouse immunoglobulin G (1:2000, 7076S, Cell signaling Technology, Danvers, MA, USA) and HRP-conjugated goat anti-rabbit immunoglobulin G (1:2000, 7074S, Cell signaling Technology, Danvers, MA, USA). The blot was stripped using Western Blot Stripping Buffer (21059, Thermo Fisher Scientific, Waltham, MA, USA) according to the manufacturer’s instructions when more than one primary antibody needed to be measured. The quantification of protein expression was analyzed by ImageJ software (National Institutes of health, Bethesda, MD, USA) and the expression of target proteins were normalized to β-actin level.

### BrdU injections

5-Bromo-20-deoxyuridine (BrdU, 50 mg/kg, B9285, Sigma, St. Louis, MO) was injected intraperitoneally at 3–6 d after tMCAO to label newly generated cells.

### Immunohistochemistry and image analysis

For immunofluorescence staining, 25-μm-thick frozen coronal brain slices were sliced on a freezing microtome. Brain slices were rinsed in PBS and 0.3% Triton X-100 in PBS (PBST), then blocked with 10% goat serum or 5% donkey serum in 0.3% PBST for 1–2 h at room temperature, followed by overnight incubation with primary antibodies (diluted in 0.3% PBST containing 1% goat or donkey serum) at 4 °C. After washing, slices were incubated with fluorochrome-conjugated secondary antibodies (diluted in 0.3% PBST containing 1% goat or donkey serum) at room temperature for 2 h. Slices were washed in 0.3% PBST three times at room temperature. Brain slices were finally mounted with DAPI Fluoromount-G (36308ES20, Yeasen, Shanghai, China). For immunostaining with mouse primary antibodies the M.O.M kit (MKB-2213, Vector Laboratories, Burlingame, CA, USA) was applied before primary antibody incubation to block nonspecific signals according to manufacturer’s instructions. Primary antibodies included: rabbit anti-IL-13R alpha1 (IL-13Rα1, 1:500, LS-C117959, LifeSpan BioSciences, Seattle, WA, USA), rabbit anti-myelin basic protein (MBP, 1:500, ab40390, Abcam, Cambridge, MA, USA), mouse non-phosphorylated anti-neurofilament H antibody (SMI32, 1:1000, 801701, BioLegend, San Diego, CA, USA), rabbit anti-nav1.6 antibody (1:300, ASC-009 Alomone, Jerusalem, Israel), mouse anti-caspr antibody (1:300, MABN69, Millipore, Billerica, MA, USA), goat anti-CD206 (1:200, AF2535,R&D Systems, Minneapolis, MN, USA), rat anti-CD16/32 (1:200, 553142, BD, San Jose, CA, USA), rabbit anti-Iba1 antibody (1:1000, 019-19741, Wako, Richmond, VA, USA), mouse anti-APC antibody (1:300, OP-80, Millopore, Billerca, MA, USA), rat anti-BrdU antibody (ab6326, Abcam, Cambridge, MA, USA) and rabbit anti-NG2 antibody (1:300, AB5320, Millipore, Billerica, MA, USA). Secondary antibodies were purchased from Jackson ImmunoResearch Laboratories (West Grove, PA, USA) and were diluted 1:1000. Secondary antibodies included: anti-rabbit secondary antibody conjugated with Alexa Flour 488 (711-545-152), anti-rabbit secondary antibody conjugated with Cy3 (711-165-152), anti–rabbit secondary antibody conjugated with Cy5 (711-605-152), anti-rat secondary antibody conjugated with Cy3 (712-165-153), anti-rat secondary antibody conjugated with Alexa Flour 488 (112-545-003), anti-mouse secondary antibody conjugated with Cy3 (115-165-146), and anti-goat secondary antibody conjugated with Alexa Flour 488 (705-545-147). The images were captured by a Nikon A1 confocal microscope and analyzed by ImageJ software (National Institutes of health, Bethesda, MD, USA).

Imaris software was used to reconstruct 3-dimensional (3D) images of confocal z-stacks. Briefly, a region of interest was selected, and the absolute intensity of each source channel was used for reconstruction and the immunosignal of each channel was remodeled to 3D images by the surface operation. Smoothing was set at 0.62 μm for all channels and images. A threshold was set to differentiate the target signal from background. Non-specific signals were then removed, and the 3D-rendered images were constructed. All images were processed with the same adjustments.

### Compound action potential measurements

Compound action potentials (CAPs) were recorded in the external capsule (EC) 35 days after tMCAO or sham operation as previously described [21]. To record CAPs, electrical stimuli were applied (bipolar stimulating electrode) across the corpus callosum (CC) at approximately 0.9 mm laterally from midline. CAPs were recorded with a glass microelectrode (filled with artificial cerebrospinal fluid, resistance 5–8 MΩ) placed in the EC 1 mm lateral to the stimulating electrode. A range of stimulation currents (0.25 mA intervals from baseline, up to 2 mA) were given to elicit CAPs in the EC. The amplitudes of the N1 (representing myelinated fibers) and N2 (representing unmyelinated fibers) components of the CAPs were measured by pClamp 10 software (Molecular Devices, San Jose, CA, USA).

### Real-time PCR

Total RNA was extracted from mouse brain tissue samples around the infarcted zone using TRIzol reagent (19201ES60, Yeasen, Shanghai, China), and then first-stand cDNA was generated using reverse transcriptase-PCR with the First Strand cDNA Synthesis Kit (K1622, Thermo Fisher Scientific, Pittsburgh, PA, USA) following the manufacturer’s instructions. Primers sequences were as follows:

5'-CTCCATGAGCTTTGTACAAGG-3' (forward) and.

5'-TGCTGATGTACCAGTTGGGG-3' (reverse) for *IL1β*,

5'-GACCCTCACACTCAGATCATCTTCT-3' (forward) and.

5'-CCTCCACTTGGTGGTTTGCT-3' (reverse) for *TNF-α,*

5'-ACACATGTTCTCTGGGAAATC-3' (forward) and.

5'-AGTGCATCATCGTTGTTCATA-3' (reverse) for *IL6*,

5'-TTTGGACACCCAGATGTTTCAG-3' (forward) and.

5'-GTCTTCCTTGAGCACCTGGATC-3' (reverse) for *CD16*;

5'-CCAAGACGATCTCAGCATCA-3' (forward) and.

5'-TTCTGGCTTGCTGAATCCTT-3' (reverse) for *CD11b*,

5'- GACCGTTGTGTGTGTTCTGG -3' (forward) and.

5'-GATGAGCAGCATCACAAGGA-3' (reverse) for *CD86*,

5'-CAAGGAAGGTTGGCATTTGT-3' (forward) and.

5'-CCTTTCAGTCCTTTGCAAGC-3' (reverse) for *CD206*,

5'-TGCGCTTGCAGAGATTAAAA-3' (forward) and.

5'-CGTCAAAAGACAGCCACTCA-3' (reverse) for *TGF-β*,

5'-CAGGGTAATGAGTGGGTTGG-3' (forward) and.

5'-CACGGCACCTCCTAAATTGT-3' (reverse) for *Ym1/2*,

5'-TCACCTGAGCTTTGATGTCG-3' (forward) and.

5'-CTGAAAGGAGCCCTGTCTTG-3' (reverse) for *Arg1*,

5'-CCTGGCTCTTGCTTGCCTT-3' (forward) and.

5'-GGTCTTGTGTGATGTTGCTCA-3' (reverse) for *IL-13.*

5'-CTGCCCAGAACATCATCCCT-3' (forward) and.

5'-TGAAGTCGCAGGAGACAACC-3' (reverse) for *Gapdh*.

Real-time qPCR was conducted on LightCycler 480 (Eppendorf, Hamburg, Germany) with HiffTM QPCR SYBR® Green Master Mix (11201ES08, Yeasen, Shanghai, China) as the detection dye. The reaction was performed at 95 °C for 5 min, 40 cycles of 95 °C for 10 s, 55 °C for 20 s and 72 °C for 20 s, followed by the melting curve. The values were the mean of triplicate assays, and the relative amount of mRNA was normalized to *Gapdh* level.

### Flow cytometry

Peripheral immune cell infiltration was analyzed by flow cytometry 3 days after tMCAO or sham operation. Mice were euthanized and peripheral blood was obtained by cardiac puncture. Mice were perfused with cold Hank's balanced salt solution (HBSS), then the ipsilateral hemisphere and spleen were harvested. The ipsilateral hemisphere was dissected into small pieces and dispersed into a single cells suspension using Neural Tissue Dissociation Kit (Trypsin) (Miltenyi Biotech, Bergisch Gladbach, Germany) by gentleMACS™ Octo Dissociator with Heaters (130-093-231, Miltenyi Biotech, Bergisch Gladbach, Germany) according to the manufacturer’s instructions. The single-cell suspensions were filtered through a 70 μm membrane and prepared for flow cytometry following our previously published protocol [19]. Briefly, single-cell suspensions were diluted to generate a 30% Percoll suspension and slowly overlaid on 70% Percoll (GE Healthcare BioSciences, Piscataway, NJ, USA). After centrifugation (800 *g*, 30 min, 18 °C) on the 30–70% Percoll gradient, the single-cell suspensions were at the interface. After being washed with HBSS containing 1% fetal bovine serum (FBS) (Sigma-Aldrich, St. Louis, MO), the single-cell suspensions were blocked on ice with anti-CD16/32 antibody (16-016-86, Thermo Fisher, eBioscience, Pittsburgh, PA) for 10 min and then stained with fluorophore-labeled antibodies at 4 °C for 30 min in the dark. The fluorophore-labeled antibodies included: (1) anti-CD45-eFluor450 (48-0451-82, Thermo Fisher, eBioscience, Pittsburgh, PA), anti-CD11b-allophycocyanin (APC)-cy7 (47-0112-82, Thermo Fisher, eBioscience, Pittsburgh, PA), anti-CD3-APC (17–0032-82, Thermo Fisher, eBioscience, Pittsburgh, PA), anti-CD11c-PerCP-cy5.5 (45–0114-82, Thermo Fisher, eBioscience, Pittsburgh, PA), anti-Gr1-PE (12-9669-82, Thermo Fisher, eBioscience, Pittsburgh, PA), anti-Ly6c-BV605 (563011, BD, San Jose, CA, USA), anti-CD19-FITC (11-0193-82, Thermo Fisher, eBioscience, Pittsburgh, PA) (2) anti-CD45-eFluor450, anti-CD11b-APC-eFluor780, anti-β-tubulin-Percp-cy5.5 (801215, BioLegend, San Diego, CA, USA), anti-O4-APC (130-119-155, Miltenyi Biotec, Bergisch Gladbach, Germany), anti-IL-13Rα1-PE (ab275599, Abcam, Cambridge, MA, USA), anti-IL-13Rα2-FITC (ab275601, Abcam, Cambridge, MA, USA). For intracellular staining, samples were permeabilized and fixed with a fixation/permeabilization solution kit (554714, BD, San Jose, CA, USA) first.

The spleen and blood were prepared following our previously published protocol [19]. The spleen was ground and filtered through a 70-μm cell membrane. Ammonium-Chloride-Potassium (ACK) lysing buffer (A1049201, Thermo Fisher, Gibco, Pittsburgh, PA) was used for five minutes in the dark to deplete red blood cells in the blood and spleen after centrifugation (500 g, 5 min, 18 °C). After washing with HBSS containing 1% FBS, the single-cell suspensions were fixed and permeabilized with Foxp3/transcription factor staining buffer set (00-5523-00, Thermo Fisher, eBioscience, Pittsburgh, PA) at 4 °C for 30 min in the dark, and then stained with fluorophore-labeled antibodies including: (1) anti-CD3-APC, anti-CD19-FITC, anti-CD4-eFluor 450 (48-0041-82, Thermo Fisher, eBioscience, Pittsburgh, PA), anti-CD8-BV510 (563068, BD, San Jose, CA, USA), anti-CD25-APC-cy7 (56-0251-82, Thermo Fisher, eBioscience, Pittsburgh, PA), anti-FoxP3-PE (12–4776-42, Thermo Fisher, eBioscience, Pittsburgh, PA) (2) anti-CD3-APC, anti-CD4-eFluor 450, anti-IFN-γ-PE (505808, BioLegend, San Diego, CA, USA), anti-IL-4-FITC (11–7042-82, Thermo Fisher, eBioscience, Pittsburgh, PA), anti-IL-17A-Percp-cy5.5 (45-7177-82, Thermo Fisher, eBioscience, Pittsburgh, PA) (3) anti-CD11b-APC-cy7, anti-CD11c-Percp-cy5.5, anti-CD117-BV605 (563146, BD, San Jose, CA, USA), anti-CD3-APC, anti-Ly6G-PE, anti-NK1.1-FITC (553164, BD, San Jose, CA, USA), anti-Siglec-F-BV421 (565934, BD, San Jose, CA, USA).

Bronchoalveolar lavage fluid was prepared following a published protocol [22]. ACK lysing buffer (A1049201, Thermo Fisher, Gibco, Pittsburgh, PA) was used for two minutes in the dark to deplete red blood cells in the bronchoalveolar lavage fluid after centrifugation (500 *g*, 5 min, 18 °C). After washing with HBSS containing 1% FBS, the single-cell suspensions were stained with fluorophore-labeled antibodies to the cell surface antigens at 4 °C for 30 min in the dark. The fluorophore-labeled antibodies included: anti-CD11c-Percp-cy5.5, anti-Siglec-F-BV421, anti-CD11b-APC-cy7, anti-Ly6G-PE, anti-CD3-APC, and anti-CD19-FITC.

Appropriate isotype controls were used according to manufacturer’s instructions (Thermo Fisher eBioscience, Pittsburgh, PA, USA). Fluorochrome compensation was performed with single-stained OneComp eBeads (Thermo Fisher eBioscience, Pittsburgh, PA, USA). Flow cytometry was performed on a Beckman Coulter CytoFLEX flow cytometer (Beckman Coulter, CA, USA), and data were analyzed by FlowJo software (Ashland, OR, USA).

### Transmission electron microscopy

Ultrastructural observation of white matter demyelination was performed by transmission electron microscopy (TEM) as described previously [23, 24]. Briefly, mice were deeply anesthetized with isoflurane (5%) and their brains were quickly removed 35 days after tMCAO. Coronal sections were sliced at 350 µm using a concussion microtome and fixed in 2.5% glutaraldehyde prepared in phosphate buffer solution for 24 h. These callosal slices matched those slices used for CAPs assessment and the target area in the CC used for TEM matched the electrophysiology recording site on the CC for CAPs recording. Regions of interest within the CC was rinsed with 0.1 M phosphoric acid and then fixed with 1% osmium acid for 2–3 h. Then the CC was dehydrated, embedded, solidified, sliced with a Leica LKB-1 ultrathin microtome at 50–60 nm, and finally stained with lead citrate. The images were captured by a Philips CM120 electron microscope (Royal Dutch Philips Electronics Ltd, Amsterdam, Holland, The Netherlands) and analyzed using ImageJ software. Four to five images (600 μm^2^ each) from each mouse were analyzed to count the ratio of myelinated and unmyelinated fibers. At least 250 random axons per mouse were analyzed to calculate the g-ratio (inner axonal diameter/outer axonal diameter). Increased g-ratio indicates a thinner myelin sheath of myelinated axons.

### Cell culture and treatment

#### In vitro primary microglia, oligodendrocyte precursor cells (OPCs) and oligodendrocytes (OLs) cultures

Primary mixed glial cell cultures were prepared from mouse/rat as described previously [25, 26]. Briefly, whole brains of 1-day-old C57BL/6J mice or Sprague Dawley rat pups were separated into single-cell suspensions by Trypsin (0.01%) at 37 °C for ten minutes and then placed in a culture flask coated with 0.01% poly-D-lysine (PDL, P-0899, Sigma-Aldrich, St. Louis, MO). Cells were cultured in Dulbecco’s modified Eagle’s medium/Nutrient Mixture F-12 Ham (DMEM/F12, 11320-033, Gibco, California, USA) supplemented with 10% FBS (10099141C, Gibco, California, USA), 1% penicillin–streptomycin (15140-122, Gibco, California, USA), 1 mM Sodium pyruvate (P2256, Sigma-Aldrich, St. Louis, MO) and 2 mM glutaMAX (35050-061, Gibco, California, USA) for 9–12 days in a humidified 37 °C incubator with 5% carbon dioxide and 95% oxygen with medium changed every 2 days. Microglia were separated from astrocytes by shaking the flasks for 1 h at 180 rpm and 37 °C. The enriched microglia were seeded in PDL-coated plates or transwell (3450, Corning Costar, Cambridge, MA) and cultured in DMEM/F12 medium for 3–5 days for use. Then, the flasks were refilled with fresh media and were subjected to shaking at 200 rpm 12–14 h to separate OPCs from the firmly attached astrocyte layer. The enriched OPCs were seeded in Poly-DL-ornithine hydrobromide (PDL-O, P0421, Sigma-Aldrich, St. Louis, MO)-coated plates and cultured in basal defined medium (BDM) containing 20 ng/mL PDGF (315-17, Peprotech, Rocky Hill, NJ, USA) and 20 ng/mL bFGF (450–33, Peprotech, Rocky Hill, NJ, USA) for 3–6 days for use. BDM is consist with Dulbecco’s modified Eagle’s medium (DMEM, 11,965–092, Gibco, California, USA), 1% penicillin–streptomycin (15140-122, Gibco, California, USA), 1 mM Sodium pyruvate (P2256, Sigma-Aldrich, St. Louis, MO), 0.1% bovine serum albumin (B2064, Sigma-Aldrich, St. Louis, MO), 50 μg/mL human apo-transferrin (T2036, Sigma-Aldrich, St. Louis, MO), 50 μg/mL insulin (I6634, Sigma-Aldrich, St. Louis, MO), 30 nM sodium selenite(S5261, Sigma-Aldrich, St. Louis, MO), 10 nM D-biotin(D4501, Sigma-Aldrich, St. Louis, MO) and 10 nM hydrocortisone (H0888, Sigma-Aldrich, St. Louis, MO). For oligodendrocyte induction, OPCs were stimulated with T3 (100 ng/mL) + CNTF (20 ng/mL) for 4 days.

#### In vitro primary neuronal cultures

Primary cortical hippocampal neurons were taken from 17-d-old C57BL/6 J mouse embryos as described previously [27]. Briefly, PDL-coated 6-well plates were seeded with 1 million cells per well. Cells were cultured in neurobasal medium (21103-049, Gibco, California, USA) supplemented with B27 (17504-044, Gibco, California, USA) for 9–10 days in a humidified 37 °C incubator with 5% carbon dioxide and 95% oxygen with one-half of the medium changed every 2 days.

#### Oxygen–glucose deprivation/reoxygenation (OGD/R)

OGD/R models were established with D-glucose-free DMEM (11966-025, Gibco, California, USA) and incubated at 37 °C in a hypoxic incubator (95% nitrogen and 5% carbon dioxide) for 1 h to simulate OGD damage. Primary neurons, OPCs and OLs were then transferred back to normal glucose-containing DMEM medium in a normal incubator for an additional 24 h (reoxygenation).

### Cell viability assay

After 24 h of reoxygenation, the primary neuron supernatant was centrifuge at 10,000 rpm for five minutes to remove cell debris and then lactate dehydrogenase (LDH) release was measured from damaged cells into the culture medium. Cell viability was measured using a LDH monitoring kit (11644793001, Roche, Mannheim, Germany) following the manufacturer’s instructions. All samples were assayed by an investigator blinded to experimental treatment conditions.

### Live/dead cell imaging

Live (green) and dead (red) staining of cultured OPCs and OLs were measured using a live/dead cell imaging kit (R37601, invitrogen, Carlsbad, CA, USA) according to the manufacturer’s instructions.

### Single-cell RNA sequencing data analysis

GSE171169 dataset (5d after tMCAO) collected from the GEO database, was loaded into the Seurat package (v4.0.6) for analysis [28]. Samples were merged without integration. Cells ranging from 500 to 5000 detected genes per cell with less than 5% mitochondrial gene expression were included in this study. The remaining cells were then log-normalized, scaled and centered using the ScaleData function. Two thousand highly variable genes which were identified by variance stabilizing transformation method were used as the input for principal component analysis (PCA). Based on the percentage of variance explained by each component that was evaluated using the Jackstraw test, the first 30 principle components were used for clustering and t-distributed Stochastic Neighbor Embedding (tSNE) analysis. Clusters were determined using the FindNeighbours and the FindClusters functions with a resolution of 0.4. Then, differential expressed genes (DEGs, log2fold change >|0.25|) of each of the resulting 15 clusters were calculated using the Wilcoxon rank sum test. Each cluster was manually annotated based on these DEGs. Microglia that include 3 clusters were then extracted as a subset for further analysis. DEGs between MG 3 and the other two clusters were calculated using Wilcoxon rank sum test with the FindMarkers function (log2fold change >|0.25|) and were further filtered based on a threshold of adjusted *P* value < 0.05. These genes were then used as an input for KEGG analysis using ClusterProfiler package [29]. Also, the original expression counts matrix of microglia subset were used as an input for GSVA using GSVA package [30]. GSVA enrichment scores as well as the expression of genes contained in JAK–STAT signaling pathway (mmu04630) were visualized using the pheatmap package. Bar plots were generated using the ggplot2 package. In additions, DEGs (adjusted *P* value < 0.05, log2fold change ≠ 0) between groups expressing IL-13Rα11 or not were used to perform KEGG pathway gene set enrichment analysis as implemented in ClusterProfiler. All the analysis of DEGs only tested genes that are detected in at least 10% of either of the two populations.

### Statistical analysis

Data are presented as mean ± standard error of the mean (SEM). The results were analyzed using GraphPad Prism software (version 8.0, La Jolla, CA, USA). Gaussian distribution was tested using the Kolmogorov–Smirnov test. Data that conform to a Gaussian distribution were analyzed by parametric tests, while non-Gaussian distributed data were analyzed by nonparametric tests. The two-tailed unpaired Student’s t test was used for comparison of two groups of Gaussian distributed data. The Mann–Whitney U rank sum test was used for non-Gaussian distributed data. The differences in means among multiple groups of Gaussian distributed data were analyzed using ordinary one-way analysis of variance (ANOVA; F-test) followed by Bonferroni’s multiple comparisons, unless noted otherwise and the Kruskal–Wallis test was used for non-Gaussian distributed data. Differences in means among groups with repeated measurements over time were analyzed by two-way ANOVA followed by Bonferroni’s multiple comparisons. Linear regression analysis was performed using SPSS software (version 20, SPSS, Inc, Chicago, IL, USA). Pearson correlation analyses were used to test correlations. In all analyses, *p* < 0.05 was considered statistically significant.

## Results

### Intranasal post-delivery of IL-13 improves neuron loss and exerts a long-term neuroprotective effect after the tMCAO

NeuN immunofluorescence staining was measured to assess neuron loss in the acute phase of stroke. Interestingly, IL-13 significantly improved the decrease of neuronal density caused by tMCAO in the peri-infarct striatum and cortex, but not in the infarct core (Additional file 1: Fig. S1A) 3 days after tMCAO, demonstrating that IL-13 treatment significantly improves the neuron loss of the peri-infarct are in the acute phase of ischemia–reperfusion injury in mice. Consistently, sensorimotor function is an important measurement for assessing neurological recovery after ischemic stroke. However, few studies have examined the effects of IL-13 on long-term outcomes after ischemic stroke. To evaluate the long-term effects of IL-13 on neurological deficits caused by tMCAO, sensorimotor function was assessed by the corner test (Fig. 1Ba) and the rotarod test (Fig. 1Bb) up to 35d after tMCAO (Fig. 1A). IL-13 treatment did not affect sensorimotor function in sham-operated mice under physiological conditions (Fig. 1Ba, b). Stroke induced severe sensorimotor impairment in mice, which was manifested as increased number of left turns in the corner test (Fig. 1Ba) and decreased latency to fall off the rotating rod (Fig. 1Bb). Interestingly, IL-13 showed a protective effect on sensorimotor dysfunction until 35 days after tMCAO, especially in the acute phase of stroke (3, 5 days after tMCAO), as shown by increased latency to fall off the rotating rod (Fig. 1Bb) and decreased number of left turns in the corner test (Fig. 1Ba). Those results demonstrated that IL-13 treatment significantly improves the neuron loss in the acute phase of ischemia–reperfusion injury in mice, and then exerts a long-term neuroprotective effect after the injury.

### Intranasal post-delivery of IL-13 promotes white matter integrity after ischemic stroke

White matter injury, characterized by demyelination and/or axonal damage, is a key component of the pathophysiological processes involved in ischemic stroke, and contributes significantly to long-term sensorimotor and cognitive deficits [31, 32]. We used double-immunostaining for MBP (a marker of myelin) and SMI32 (a marker of non-phosphorylated neurofilaments, usually for detecting the demyelinated axons) to evaluate structural white matter integrity 35 days after tMCAO (Fig. 1C, D). As expected, immunofluorescence staining results showed that tMCAO mice displayed a decrease in the fluorescence intensity of MBP and a significant increase in the SMI32/MBP ratio in the white matter-rich external capsule (EC) and striatum (STR) around the infarcted zone compared to sham mice (Fig. 1C), indicating axonal demyelination after tMCAO. Furthermore, IL-13 treatment increased MBP intensity, decreased the SMI32/MBP ratio, and increased the MBP^+^ area of the CC compared to vehicle-treated mice 35 days after tMCAO (Fig. 1C, D), indicating that IL-13 treatment enhances white matter integrity by reducing tMCAO-induced loss of myelin protein and axonal demyelination. Additionally, immunostaining with MBP and SMI32 3 days after tMCAO showed that IL-13 did not reduce the demyelination damage caused by tMCAO during the acute phase of stroke (Additional file 1: Fig. S1B), suggesting that IL-13 may promote white matter repair after tMCAO.

Clinically, loss of white matter integrity is strongly linked to poor stroke outcomes in ischemic stroke patients [33]. To ascertain whether behavioral improvement induced by IL-13 was related to improvement in white matter integrity after tMCAO, we used a series of linear regression analysis to assess whether there was a correlation between behavioral performance and white matter integrity 35 days after tMCAO. According to our results (Fig. 1E), SMI32/MBP ratio in the STR was negatively correlated with the latency to fall in the rotarod test (Fig. 1E, upper panel). Dots of different color represent 4 group of mice ( black, blue, red, and green dots for Sham + veh, Sham + IL-13, tMCAO + veh, and tMCAO + IL-13, respectively) in the figure. MBP fluorescence intensity in the STR was negatively correlated with the number of left turns in the corner test, while positively correlated with the latency to fall in the rotarod test (Fig. 1E, down panel). Additionally, there were significant positive correlations between the MBP^+^ area in CC and the latency to fall in the rotarod test (Fig. 1E, down panel). Thus, the improvement in white matter integrity with IL-13 treatment contributed to the sensorimotor improvement after tMCAO.

**Fig. 2:**
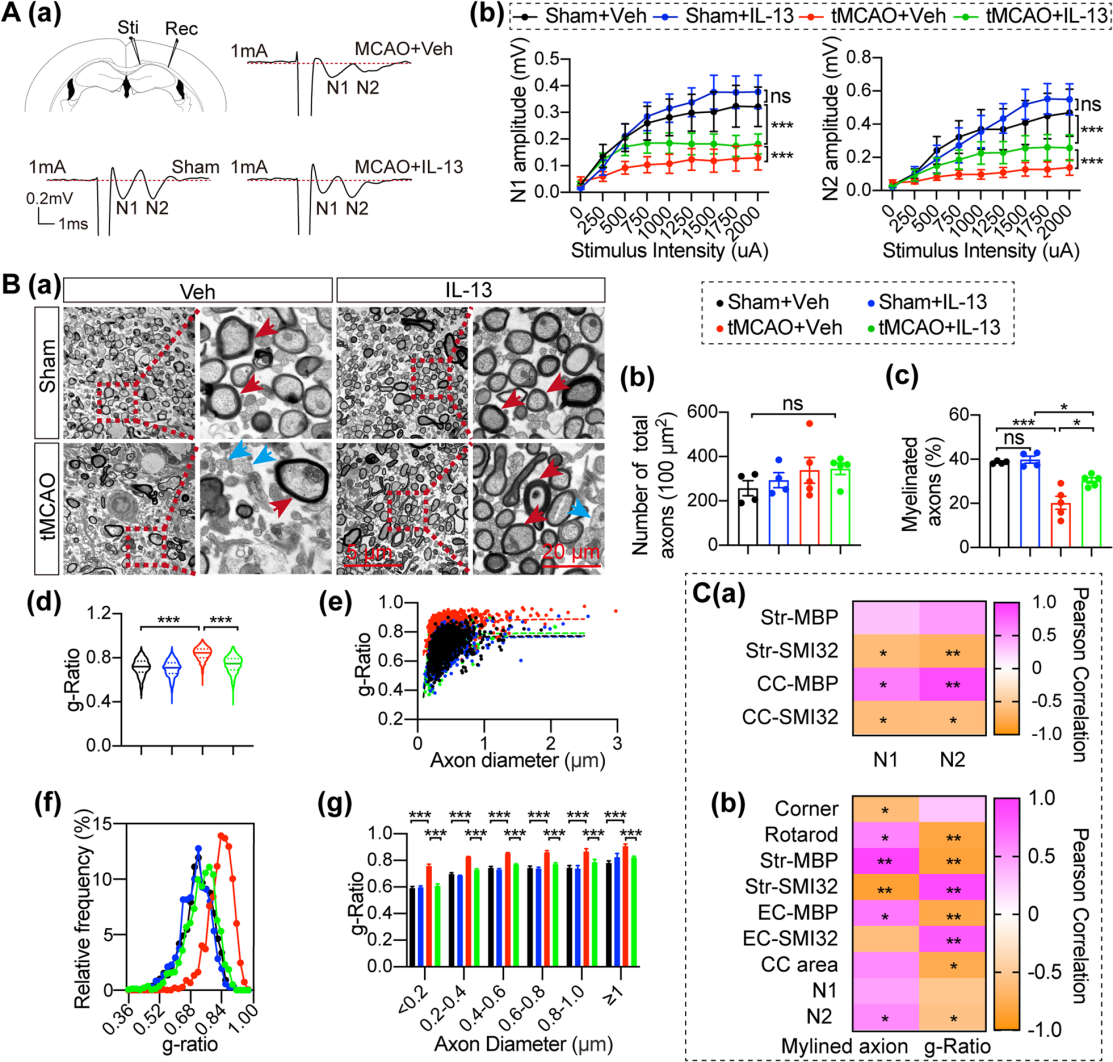
Intranasal IL-13 treatment enhances functional white matter integrity 35 days after tMCAO. **A** CAPs were recorded in the EC 35 days after tMCAO or sham operation **Aa** Diagram of the location of the stimulating and recording electrodes with 1 mm distance from the stimulus site to the recording site at the EC and the representative trace of the CAPs. **Ab** Comparison of peak-to-peak N1 an N2 amplitudes after various stimulation intensities *n* = 6–8/group. **Ba** Representative electron micrographs. The red arrows indicate myelinated axons, and blue arrows indicate unmyelinated axons. Scale bar: 5 μm. Scale bar = 20 μm for enlarged view. **Bb**, **c** Quantification of the number of total axons and the percentage of myelinated axons in electron micrographs *n* = 4–5/group. **Bd** Violin plot of the g-ratio, **Be** scatter plots of the g-ratio, and **Bf** frequency histogram of the g-ratio as a function of total axon diameter. **Bg** Comparison of g-ratio as a function of axon diameter. Quantification of myelinated axons and myelin thickness was done in five representative images/animals *n* = 1097 axons from 4 animals for Sham + Veh; *n* = 1089 axons from 4 animals for Sham + IL-13; *n* = 1345 axons from 5 animals for tMCAO + Veh; n = 1334 axons from 5 animals for tMCAO + IL-13 **Ca** Pearson correlation between the MBP/SMI32 immunostaining, and CAPs amplitudes at 1 mA stimulation intensity. *n* = 2–4/group. **Cb** Pearson correlation between the animals’ performance, CAPs amplitudes at 1 mA stimulation intensity, MBP/SMI32 immunostaining, and electron microscopy index *n* = 2–5/group. All data are presented as the mean ± SEM. *p ≤ 0.05, **p ≤ 0.01, ***p ≤ 0.001. Two-way ANOVA followed by Bonferroni’s post hoc (**Ab**, **Bg**), One-way ANOVA followed by Bonferroni’s post hoc (**Bb**, **c**), Kruskal–Wallis test followed by Dunn’s post hoc (**Bd**), and Pearson Correlation (**C**)

CAPs are helpful for evaluating impairment in white matter function. CAPs show two peaks, including the early peak (N1), which represents the fast-conduction of myelinated fibers, and the later peak (N2) representing the slow-conduction of unmyelinated fibers (Fig. 2Aa). We found that IL-13 treatment did not influence axon transmission in sham-operated mice under physiological conditions. Consistent with our previous findings [34], the amplitudes of N1 and N2 were significantly decreased after tMCAO (Fig. 2Ab), suggesting injury to both myelinated and unmyelinated fibers after tMCAO. Furthermore, mice treated with IL-13 showed slight improvement in the N1 and N2 amplitudes (Fig. 2Ab) compared to vehicle-treated mice after tMCAO, indicating that IL-13 treatment significantly attenuated demyelination of myelinated fibers and the damage to unmyelinated fibers after tMCAO.

TEM was performed 35d after tMCAO to verify the ultrastructural changes in the EC and to determine whether IL-13 improved axonal myelination (Fig. 2B). There was no difference in total axon density among the four groups (Fig. 2Bb), but the percentage of myelinated fibers decreased significantly after stroke but improved significantly after IL-13 treatment (Fig. 2Bc). The violin plots of the g-ratio (Fig. 2Bd), scatter plots of the g-ratio (Fig. 2Be), and frequency histogram of the g-ratio (Fig. 2Bf) all revealed a significant increase of the g-ratio after tMCAO and a significant decrease of g-ratio after IL-13 treatment. Meanwhile, IL-13 treatment significantly reduced the tMCAO-induced increase in the g-ratio across different axon diameters suggesting that IL-13 maintained axonal myelination or promoted axonal remyelination after stroke (Fig. 2Bg).

Similarly, we compared the behavioral results 35 days after tMCAO and the immunofluorescence staining of MBP/SMI32 with the amplitudes of the N1and N2 of CAPs (Fig. 2Ca) or microstructure of the EC (Fig. 2Cb) performed in the same animals. A variety of behavioral and immunofluorescence staining indicators were correlated with the amplitudes of the N1/N2 of CAPs (Fig. 2Ca), the percentage of myelinated fibers and the g-ratio (Fig. 2Cb). We also found that the amplitudes of the N2 were correlated with the percentage of myelinated fibers and g-ratio (Fig. 2Cb). Together, these results demonstrate that IL-13 treatment contributes to improved white matter integrity in both structure and function, and the improvement of white matter integrity is correlated with behavioral improvement.

### Intranasal IL-13 treatment reduces inflammation but does not alter the adaptive immune cell population in brain after stroke

**Fig. 3:**
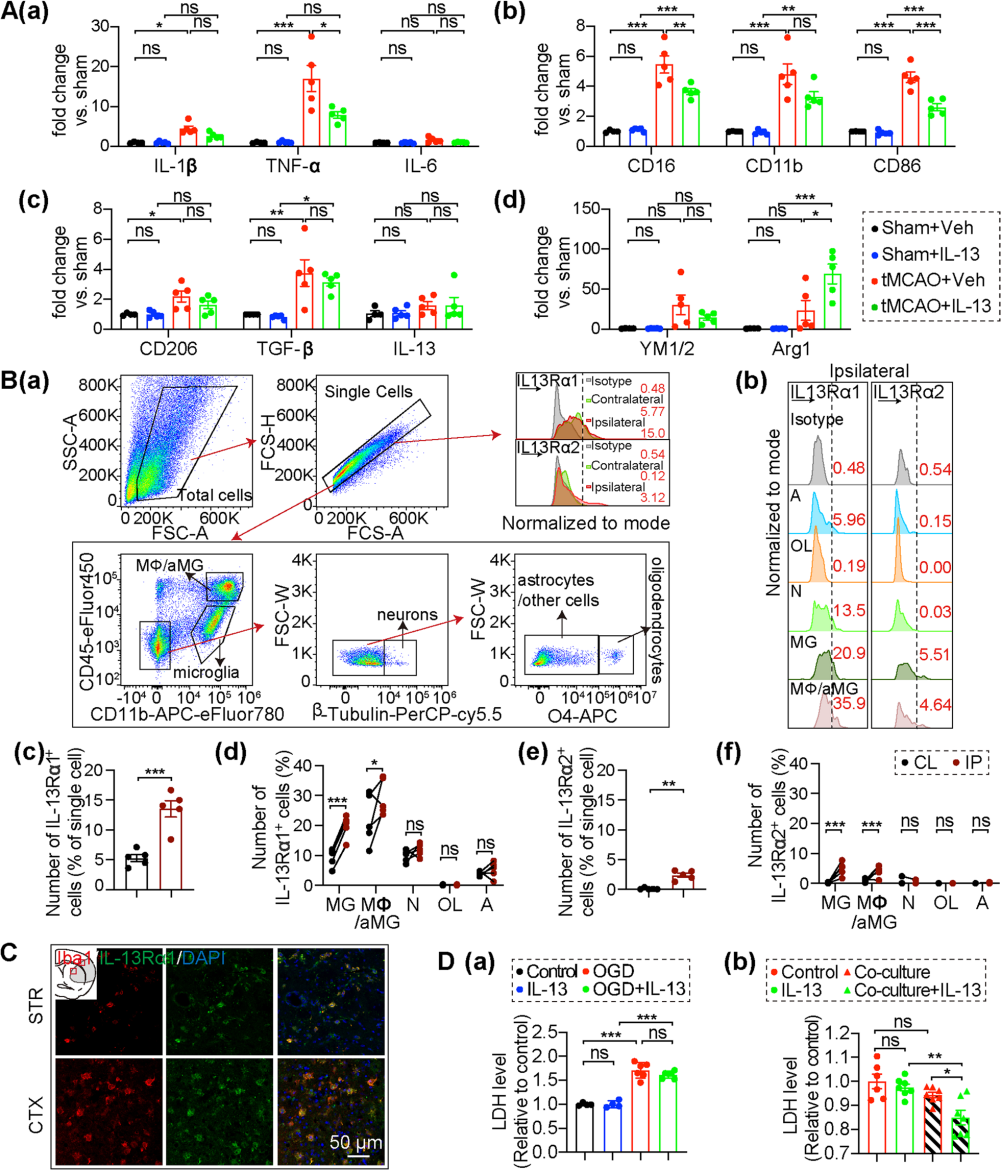
IL-13Rα1 on microglia/macrophages may play a key role in stroke brain. **A** qPCR measurement of the mRNA expression levels of pro-inflammatory markers **Aa**, **b** and anti-inflammatory markers **Ac**–**d** in the brain 3 days after tMCAO or sham operation. Data are shown as fold change of sham-vehicle controls. *n* = 4–5/group. **B** The expression of IL-13 receptors in the brain assessed by flow cytometry 3 days after tMCAO. **Ba **Gating strategy for the IL-13 receptors in the brain. **Bb** The fluorescence expression of IL-13Rα1 and IL-13Rα2 (Bc-f) observed in astrocytes/other cells (**A**), oligodendrocytes (OL), neurons (N), microglia (MG), and macrophages (MΦ/aMG). **Bc**, **d** Quantification of IL-13Rα1^+^ cells in the brain by flow cytometry. **Be**, **f** Quantification of IL-13Rα2^+^ cells in the brain by flow cytometry. *n* = 5/group. **C** Representative images of IL-13Rα1 (green) and Iba1 (red) immunostaining in the striatum 3d after tMCAO. Scale bar: 50 µm. **D** In vitro experiments. **Da** Quantification of primary neuron survival after 1 h OGD/R challenge. *n* = 4–6/group. **Db** Quantification of in vitro primary neuron survival 1 h after OGD/R in a transwell system (co-cultured with Veh/IL-13 treated primary microglia). *n* = 6–7/group. All data are presented as the mean ± SEM. *p ≤ 0.05, **p ≤ 0.01, ***p ≤ 0.001. Unpaired Student’s *t*-test (**Bc**), Mann–Whitney *U* test (**Be**), two-way ANOVA followed by Bonferroni’s post hoc (**Bd**, **f**), and one-way ANOVA followed by Bonferroni’s post hoc (**A**, **D**)

Inflammation plays an important role in cerebral ischemic injury, therefore we explored whether IL-13 treatment correlated with induction of anti-inflammatory genes and/or downregulation of pro-inflammatory genes in the stroke brain. We evaluated the gene expression levels of multiple pro-inflammatory factors (IL-1β, TNF-α, IL-6, CD16, CD11b, and CD86) and anti-inflammatory factors (CD206, TGF-β, IL-13, YM1/2, and Arg1) by qPCR 3 days after tMCAO (Fig. 3A). IL-13-treated tMCAO brains showed decreased expression of multiple pro-inflammatory factors compared to vehicle-treated tMCAO brains, including TNF-α, CD16, and CD86 (Fig. 3Aa, b), with the others showing similar trends (Fig. 3Aa, b), whereas one of the anti-inflammatory factors, Arg1, was significantly upregulated (Fig. 3Ac, d). Overall, IL-13 treatment suppressed the pro-inflammatory response and promoted the anti-inflammatory response in the stroke brain.

In addition to the activation of glia, the recruitment of peripheral immune cells is also a feature of neuroinflammation [35]. We detected the infiltration of peripheral immune cells in the brain 3 days after tMCAO by flow cytometry analysis using the gating strategy of CD3^+^ T cells, CD19^+^ B cells, dendritic cell (CD11b^+^CD11c^+^), neutrophils (Gr1^+^CD11b^+^CD45^+^ CD11c^−^, macrophages (CD45^high^CD11b^+^, also including some activated microglia, MΦ/aMG), microglia (CD11b^+^CD45^low^), and inflammatory macrophages (CD45^high^CD11b^+^Ly6C^+^, also including some activated microglia, MΦ/aMG) [36] (Additional file 1: Fig. S2Aa). Our results showed that the number of multiple infiltrating peripheral immune cells (T cell, macrophage and inflammatory macrophage) were increased in brain 3 days after tMCAO, but there was no significant difference in the number of various infiltrating lymphocytes between vehicle-treated tMCAO mice and IL-13-treated tMCAO mice (Additional file 1: Fig. S2Ab-h, S2C). Although other immune cells were also present, as expected, the largest population of immune cells in the stroke brain were microglia (Additional file 1: Fig. S2B).

In addition, studies on peripheral inflammatory diseases have found that IL-13 plays a regulatory role through neutrophils, mast cells, natural killer cells, and eosinophils, etc. [37, 38]. We first evaluated the effect of IL-13 on the subsets of lymphocyte in the blood (Additional file 1: Fig. S3B) and spleen (Additional file 1: Fig. S3C) 3 days after tMCAO by flow cytometry analysis using the gating strategy of the CD19^+^ B cells, CD3^+^ total T cells, CD8^+^ T cells, CD4^+^ T cells and Foxp3^+^CD4^+^ regulatory T (Treg) cells (Additional file 1: Fig. S3A). Then we evaluated the subsets of CD4^+^ T cells in the blood (Additional file 1: Fig. S3E) and spleen (Additional file 1: Fig. S3F) using the gating strategy of IFN-γ^+^ Th1 cells, IL-4^+^ Th2 cells, and IL-17^+^ Th17 cells (Additional file 1: Fig. S3D). We also evaluated the effect of IL-13 on function-related immune cells in blood, including dendritic cells, macrophages, eosinophils, neutrophils, mast cells, and natural killer (NK) cells (Additional file 1: Fig. S4A, B). Our results showed that IL-13 treatment did not alter the number of peripheral immune cells in each subset we tested in the blood (Additional file 1: Figs. S3B, E and S4B) or spleen (Fig. S3C, F) from sham or tMCAO mice. These data do not support the involvement of the peripheral immune response in the neuroprotective effect of IL-13.

It has also been reported in bronchial asthma that IL-13 induces inflammatory cell influx into the lung [39, 40]. Therefore, we also tested the effects of IL-13 on various function-related immune cells, including T cells, B cells, dendritic cells, macrophages, eosinophils, and neutrophils in the lung 3 days after tMCAO (Additional file 1: Fig. S4C, D). Our results showed that IL-13 had no effect on any function-related immune cells in lung after tMCAO.

### IL-13 administration promotes neuron survival after OGD/R in a microglia-dependent manner

Next, we explored the possible cellular mechanism of the protective effect of IL-13 after stroke. Recombinant mouse IL-13 consists of 110 amino acids with a predicted molecular weight of 12.1 kDa. It has been reported that intranasal drug delivery can pass through the blood–brain barrier into the brain parenchyma [41–44]. To confirm the delivery of IL-13 to the brain parenchyma via the intranasal route we used ELISA to detect the level of IL-13 protein in the brain three hours after IL-13 or vehicle administration at 3 days after tMCAO. Our results showed that IL-13 protein levels were significantly increased in the brains of sham and tMCAO mice that received IL-13 administration compared to vehicle (Additional file 1: Fig. S5A).

IL-13 functions are mediated via IL-13 receptors on the cell surface. Therefore, we assessed the expression of IL-13 receptors in the brain 3 days after tMCAO by flow cytometry analysis using the gating strategy of macrophages (CD45^high^CD11b^+^, including some activated microglia, MΦ/aMG), microglia (MG, CD11b^+^CD45^low^), β-tubulin^+^ neurons (N), O4^+^ oligodendrocytes (OL), and O4^−^ astrocytes/other cells (A) (Fig. 3Ba, b). We found that IL-13Rα1 expressed in healthy brain (Fig. 3Bc), while IL-13Rα2 is absent (Fig. 3Be), consistent with previous reports [45, 46]. Compared to the contralateral hemisphere, the expression of IL-13Rα1 (Fig. 3Bc, d) and IL-13Rα2 (Fig. 3Be, f) increased significantly (mainly in microglia and macrophages) in the ipsilateral hemisphere. The cells expressing IL-13Rα1 (13.544% ± 1.390) were more than five times higher than the cells expressing IL-13Rα2 (2.408% ± 0.3508) after tMCAO. Considering that IL-13Rα2 is a decoy receptor that is not involved in signal transduction, and its expression level is much lower than IL-13Rα1, we focused on IL-13Rα1 in this study. We detected IL-13Rα1 protein in the brain 3 days after tMCAO. The expression level of IL-13Rα1 increased significantly after tMCAO, while exogenous administration of IL-13 did not result in increased IL-13Rα1 protein levels (Additional file 1: Fig. S5B). Interestedly, only the number of microglia and macrophages expressing IL-13Rα1 increased significantly after tMCAO, even though IL-13Rα1 is expressed in microglia, macrophages, and neurons under normal physiological conditions (Fig. 3Bc, d). Immunofluorescence staining also showed co-labeling of IL-13Rα1 and microglia/macrophage (Iba1^+^) in the peri-infarct cortex and striatum 3 days after tMCAO (Fig. 3C), indicating the express of IL-13Rα1 on morphologically activated microglia/macrophage after stroke. Our results suggested that IL-13Rα1 might play a key role in the stroke brain, especially in microglia/macrophages. In vitro experiments in a co-culture system were used to elucidate the effect of IL-13 through the IL-13Rα1 on microglia (Additional file 1: Fig. S5C). Different concentrations of IL-13 (10–50 ng/ml) had no toxic effects on mice primary microglia (Additional file 1: Fig. S5D). In keeping with our previous study [18], we chose 20 ng/ml IL-13 for in vitro experiments. Interestedly, IL-13 (20 ng/mL) had no direct effects on primary neuron survival following OGD/R challenge in the neuron alone culture (Fig. 3Da), but IL-13 treated primary microglia and primary neuron co-cultures subjected to 1 h OGD/R showed significantly enhanced neuron survival after OGD/R compared with co-cultured primary microglia and neurons without IL-13 treatment (Fig. 3Db). Together, these results demonstrate the importance of microglia in the beneficial effects of IL-13 against OGD/R -induced neuronal death.

### IL-13 reduces OL/OPC death after OGD/R and encourages OPC differentiation into mature oligodendrocytes in a microglia-dependent manner

**Fig. 4:**
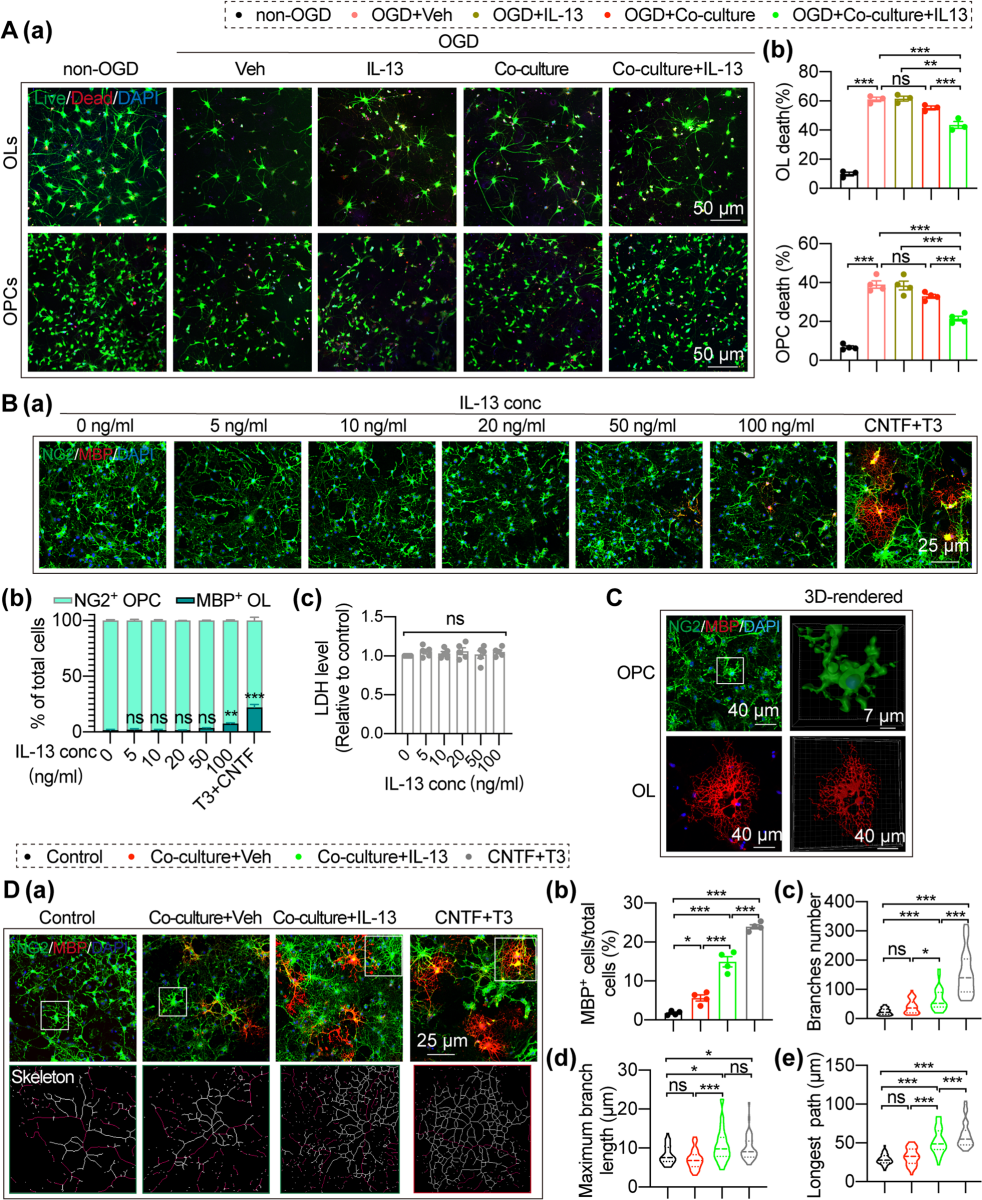
IL-13 reduces OL/OPC death after exposure to OGD/R and encourages OPC differentiation in a microglia-dependent manner. (A) OL/OPC survival after OGD/R was quantified by live/dead staining. **Aa** Representative images of live (green) and dead (red) staining of cultured oligodendrocytes or OPCs. Scale bar: 25 μm. **Ab** Percentages of dead cells were quantified. Data are from 4 independent experiments. **B**–**D** NG2 and MBP immunostaining was performed to evaluate OPC differentiation. **B** Primary OPC cultures were treated with T3 (100 ng/mL) + CNTF (20 ng/mL) or escalating concentrations of IL-13 for 3 days. **Ba** Cells were triple-stained with MBP (red), NG2 (green), and DAPI (blue). Scale bar: 25 μm. **Bb** Quantification of MBP^+^ OLs and NG2^+^ OPCs after treated with escalating concentrations of IL-13. Data are from 4 independent experiments. **Bc** Quantification of OPCs survival after treated with escalating concentrations of IL-13. *n* = 5/group. **C** Typical OPC and OL were selected for reconstruction of 3D-rendered images. **D** Primary OPCs were co-cultured with microglia treated with/without IL-13 (20 ng/ml) for 3 days. **Da** Cells were then immunostained for MBP (red) and NG2 (green), and nuclei were labeled with DAPI (blue). After skeleton reconstruction, the protrusion length and the number of protrusions of each cell were analyzed. White rectangles illustrates: where skeleton images were taken from. The red reconstruction line in the skeleton images show the longest path of each cell. Scale bar: 25 μm. **Db** Quantification of MBP^+^ OLs. Data are from 4 independent experiments. **Dc**–**e** Quantification of the number, the maximum protrusion length and the longest path of each cell. Each point shown in the results represents a cell. Four independent experimental samples were analyzed in each group, and a total of n = 40 cells were analyzed in each group. All data are presented as the mean ± SEM. *p ≤ 0.05, **p ≤ 0.01, ***p ≤ 0.001. One-way ANOVA followed by Bonferroni’s post hoc, or Kruskal–Wallis test followed by Dunn’s post hoc

The survival of preexisting oligodendrocytes and their progenitor cells is vital for white matter integrity upon insults and restoration of myelin sheaths after demyelination. In light of the potent protective effects of IL-13 on white matter (Figs. 1 and 2), we assessed its role in OL and OPC survival after exposure to OGD/R. Microglia were co-culture with the OLs or OPCs that had been subjected to 1 h OGD for 24 h and cell survival after OGD was quantified by live/dead staining (Fig. 4A, Additional file 1: Fig. S5E). The results confirmed that IL-13 (20 ng/mL) treatment on primary oligodendrocytes or OPC cultures failed to protect them against OGD/R (Fig. 4A). Interestedly, the co-culture of IL-13 (20 ng/mL)-treated microglia significantly reduced OL and OPC death after OGD/R (Fig. 4A).

Successful differentiation of OPCs into myelinating oligodendrocytes is important for remyelination after stroke [34]. Next, we investigated the impact of IL-13 on OPC differentiation, as mature OLs form the myelin sheaths. In another set of in vitro experiments, IL-13 was added directly to primary OPC cultures at increasing concentrations, from 5 to 100 ng/mL. The number of NG2^+^ OPCs and MBP^+^ mature OLs was analyzed to evaluate OPC differentiation 72 h after IL-13 administration (Fig. 4B, Additional file 1: Fig. S5Fa). Different concentrations of IL-13 (5–100 ng/ml) had no toxic effects on mice primary OPC (Fig. 4Bc). With the NG2^+^ OPC/MBP^+^ OL double staining (Fig. 4Ba, b), IL-13, the concentration was less than 100 ng/mL, failed to encourage OPC differentiation into mature oligodendrocytes, at which its efficacy was comparable to our positive control, the pro-differentiation T3 + CNTF (Fig. 4Ba, b). Interestedly, IL-13 (20 ng/mL) had no directly effects on OPC differentiation, but the same concentration of IL-13 treated primary microglia and primary OPC co-cultures (Additional file 1: Fig. S5Fb) showed significantly increased MBP^+^ mature OLs compared with co-cultured primary microglia and OPC without IL-13 treatment (Fig. 4Da (up panel), b). Interestedly, compared with the NG2^+^ OPC, MBP^+^ OL had more branches and larger cell area, as shown in the 3D-rendered images (Fig. 4C). In addition, we also carried out a skeleton reconstruction of OPC and OL, in an attempt to further evaluate the OPC differentiation by analyzing the number and length of OPC and OL branches. Compared with the control OPCs, MBP^+^OLs had more branches and larger cell area, which is manifested as increased branches number, maximum branch length, and longest path (Fig. 4Da, down panel). IL-13 treated primary microglia and primary OPC co-cultures showed significantly increased branches number (Fig. 4Dc), maximum branch length (Fig. 4Dd), and longest path (Fig. 4De) compared with co-cultured primary microglia and OPC without IL-13 treatment, suggesting that although OPCs co-cultured with IL-13 treated microglia has not yet differentiated into MBP^+^OLs, they show a cell morphology similar to mature OLs.

### JAK–STAT signaling pathway may be involved in IL-13-induced regulation of microglial activation

**Fig. 5:**
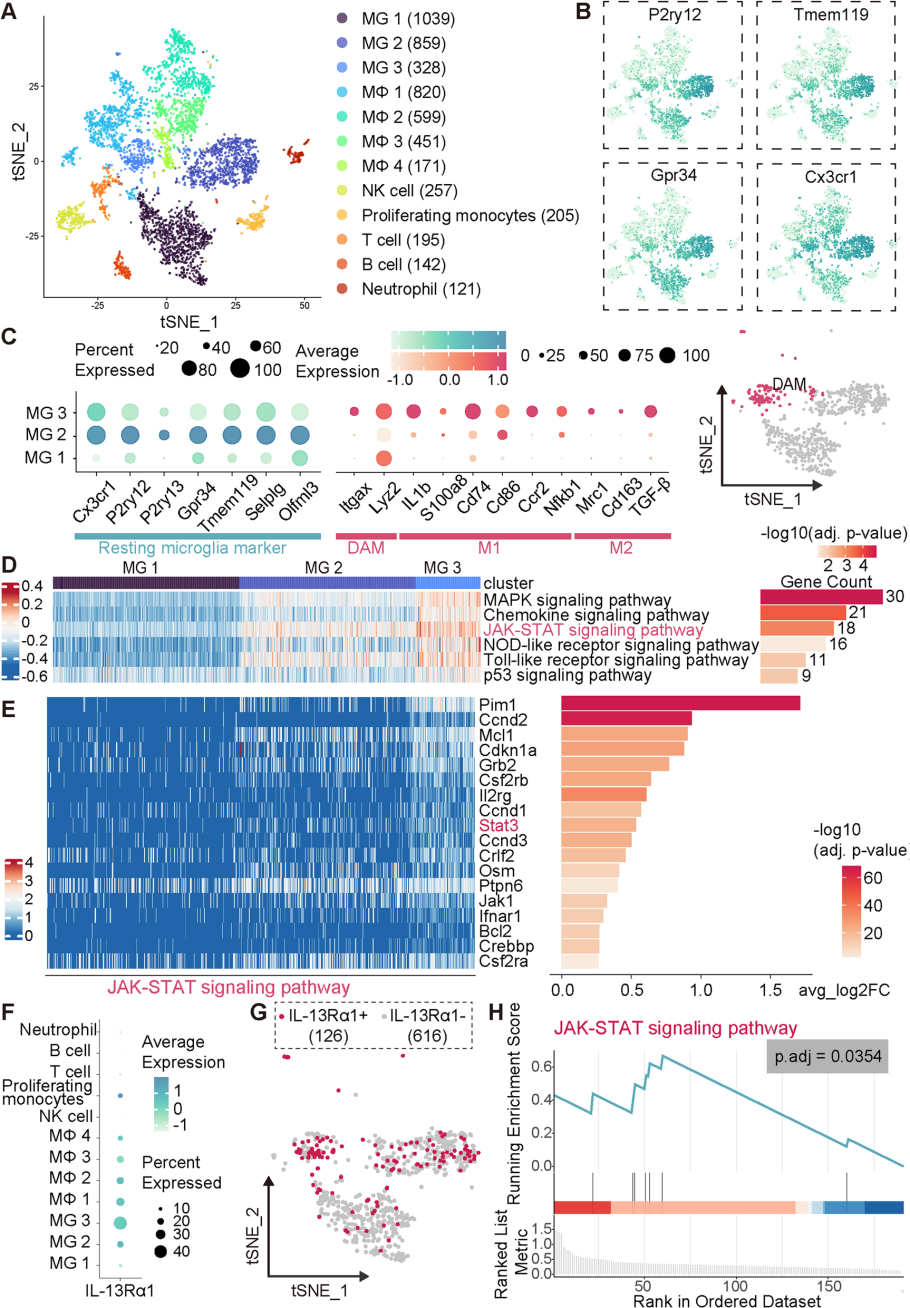
JAK–STAT signaling pathway may be involved in IL-13-induced regulation of microglial activation. **A** tSNE plot depicting identified CD45^high^ brain cells obtained from 5 days after tMCAO. **B** Expression of microglia conserved marker in tSNE plot, P2ry12, Tmem119, Gpr34, Cx3cr1. **C** Left panel, dot plot showing representative homeostatic microglia markers (Cx3cr1, P2ry12, P2ry13, Gpr34, Tmem119, Selplg, Olfml3), DAM markers (Itgax, Lyz2), M1-like phenotype markers (IL-1b, S100a8, Cd74, Cd86, Ccr2, Nfkb1) and M2-like phenotype markers (Mrc1, Cd163, TGF-β). Right panel, DAM determined accordingly were highlighted in tSNE plot of microglia subclusters. **D** Heat map showing GSVA enrichment scores per cell of microglia subclusters in five selected KEGG signaling pathways that were significantly enriched as shown in the right bar plot which visualized the results from KEGG pathway over-representation analysis using DEGs of MG 3 versus MG1&MG2 as input. **E** Heat map depicting expression of the 18 DEGs from JAK–STAT signaling pathway of each cell in **D** and their corresponding fold change. **F** IL-13Rα1 expression across all clusters. **G** tSNE plot highlighting IL-13Rα1-expressing cells. **H** GSEA running score and ranked list of DEGs (IL-13Rα1^+^ versus IL-13Rα1^−^) within JAK–STAT signaling pathway

Previous studies investigating the effect of IL-13 against stroke focused on the activation status changes of microglia/macrophages, and found that IL-13 changed the activation state of microglia/macrophages towards the protective M2-like phenotype polarized [47, 48], but the underlying mechanism remains unknown. To this end, we analyzed a publicly available single-cell RNA-seq dataset (access No. GSE171169) in which CD45^high^ immune cells were sorted from ischemic mouse brains at 5 days after tMCAO. This time point may be a critical timing for IL-13 to interfere with microglial/macrophages activation since the mRNA expression of either M1-like or M2-like markers generally reaches an obvious level at this timepoint [49]. We first identified 12 distinct clusters based on their expression profile, consisting of 6 immune cell types apart from a cluster of proliferating monocytes: microglia(MG), macrophage(MΦ), natural killer cell (NK), T cell, B cell and neutrophil (Fig. 5A). As depicted in Fig. 5B, all subtypes of microglia expressed conserved markers for microglia, including P2ry12, Tmem119, Gpr34, Cx3cr1 [50, 51].

Therefore, we further identified 3 unique subclusters of a total of 2226 microglia and 4 unique subclusters of a total of 2041 macrophages in an unsupervised manner (Fig. 5A). The scRNA-seq analysis revealed that microglia, rather than macrophages, showed highly heterogeneous expression of IL-13Rα1 in different subclusters (Fig. 5F). Of note, MG 3 cluster presented a higher frequency of IL-13Rα1 expression (Fig. 5F, G), suggesting a potential role of the IL-13/IL-13Rα1 pair in regulating microglial activation. Therefore, we reasoned that MG 3 cluster may play a predominant role in IL-13-mediated regulation of cerebral inflammation.

Compared to MG 2 cluster, MG 1/3 cluster showed lower expression of homeostatic microglia markers including Cx3cr1, P2ry12, P2ry13, Gpr34, Tmem119, Selplg, Olfml3 (Fig. 5C). This phenotype has also been reported in stroke and many other diseases [50, 52, 53], indicating the inflammatory disease-associated phenotypes of MG 1/3 cluster. Strikingly, MG 3 cluster also demonstrated upregulated expression of Itgax, Lyz2 (Fig. 5C), showing high similarity to previously reported disease-associated microglia in Alzheimer's disease (AD) [52, 54]. Notably, many more genes including both M1-like microglia markers (eg. IL-1b, S100a8, Cd74, Cd86, Ccr2, Nfkb1) and M2-like microglia markers (eg. Mrc1, Cd163, TGF-β) [49, 54–56] are also highly expressed in MG 3 cluster (Fig. 5C), indicating that MG 3 cluster have an intermediate phenotype between pro-/anti- inflammatory activation whereas no definite M1/M2-like microglia cluster was found. Therefore, we regarded MG 3 cluster as disease-associated microglia (DAM) in ischemic stroke, which represents a general activation state regardless of M1/M2-like state or a state along a spectrum from M1-like to M2-like.

To explore potential signaling pathway activated in DAM post-stroke, we performed differential expressed gene (DEG) analysis to identify DEGs (log2FC >|0.25|, p.adj < 0.05) between MG 3 cluster and the other microglia subclusters. KEGG pathway enriched for these DEGs revealed five significant signaling pathways (Fig. 5D). We also employed gene set variation analysis (GSVA) to further observe heterogeneity specific to the above five KEGG pathways of these 3 microglia subclusters in an unsupervised manner (Fig. 5D, left panel). In particularly, MG 3 cluster was found to show the overexpression of 18 genes involved in JAK–STAT signaling pathway (mmu04630) (Fig. 5E), particularly including STAT3, which contributes to stroke-associated neuroinflammation due to overactivation as validated in previous studies [17].

Interestingly, Gene Set Enrichment Analysis (GSEA) revealed that compared to IL-13Rα1-negative cells, IL-13Rα1-expressing cells were also enriched for JAK–STAT signaling pathway (Fig. 5H), yet not for the other signaling pathways aforementioned. Collectively, our findings associated the role of IL-13 with JAK–STAT signaling pathway, which may be the underlining mechanism for IL-13-induced regulation of microglia activation.

### IL-13 inhibits pro-inflammatory responses and promotes anti-inflammatory responses in microglia/macrophages by inhibiting STAT3 activation after ischemic stroke

It has been reported that the strong induction of p-STAT3 is predominantly localized to microglia/macrophages in the ischemic brain [16]. Consistent with this finding, we observed minimal protein co-expression of p-STAT3 and Iba1 in the microglia/macrophages on the contralateral side, whereas high expression was observed in microglia/macrophage in the infarcted striatum 3 days after tMCAO (Additional file 1: Fig. S6A). The majority of p-STAT3^+^ cells (94.88% ± 0.8036) were microglia/macrophages (Additional file 1: Fig. S6B). Our previous research confirmed IL-13 inhibited the expression of pro-inflammatory factors induced by lipopolysaccharide (LPS) in cultured microglia [18]. STAT3 plays a vital role in regulating microglia activation and the inflammatory response, and is a recognized regulator of inflammatory gene expression and a marker of CNS damage [17]. To ascertain whether IL-13 suppressed the inflammatory response by inhibiting STAT3 activation in the stroke brain, we firstly assess that IL-13 whether directly inhibit the STAT3 activity in vitro using rat primary microglia culture. Our results showed that IL-13 alone did not activate STAT3 in resting microglia, but did inhibit the phosphorylation of STAT3 (p-STAT3) in LPS-treated microglia, indicating that IL-13 inhibits inflammation-induced STAT3 activation in microglia (Additional file 1: Fig. S6C–E).

**Fig. 6:**
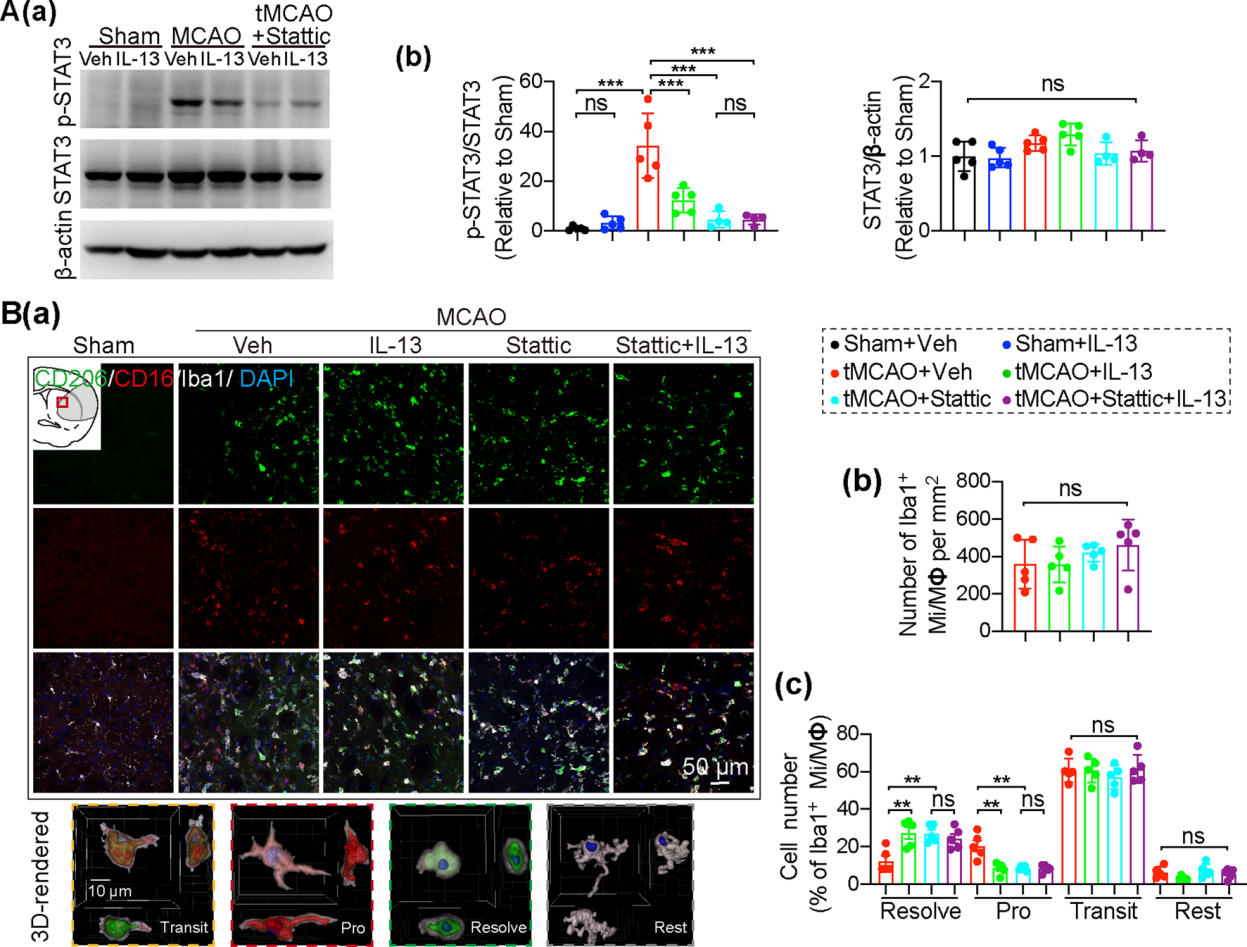
IL-13 regulates microglia/macrophages activation after stroke by inhibiting STAT3 activation. **A** Brain p-STAT3 and STAT3 expression were measured using Western blots 3d after tMCAO or sham operation. **Aa** Representative image of p-STAT3, STAT3 and β-actin. **Ab** Quantification of p-STAT3 and STAT3 expression in Western blots. *n* = 4–5/group. **B** Microglia/macrophages activation were measured 3d after tMCAO or sham operation. **Ba** Representative images of CD206 (green), CD16 (red), and Iba1 (white) immunostaining in the peri-infarct striatum. Scale bar: 50 µm. Four typical microglia were selected, and the absolute intensity of each source channel was used for reconstruction of 3D-rendered images. Scale bar: 10 µm. **Bb** Quantification of Iba1^+^ microglia/macrophages. *n* = 5/group. **Bc** Quantification of CD206^+^CD16^−^Iba1^+^ Resolve (resolution), CD16^+^CD206^−^Iba1^+^ Pro (pro-inflammatory), CD16^+^CD206^+^Iba1^+^ Transit (transitional) and CD16^−^CD206^−^Iba1^+^ Rest (resting) microglia/macrophages in the striatum. *n* = 5/group. All data are presented as the mean ± SEM. *p ≤ 0.05, **p ≤ 0.01, ***p ≤ 0.001. One-way ANOVA followed by Bonferroni’s post hoc, or Kruskal–Wallis test followed by Dunn’s post hoc

We further confirmed that IL-13 suppressed the inflammatory response by inhibiting STAT3 activation in vivo*.* As expected, p-STAT3 was minimally expressed in sham-operated mice under physiological conditions, while tMCAO caused a huge increase in p-STAT3 (Fig. 6A), which may be due to the increased inflammation caused by tMCAO. Notably, IL-13 significantly downregulated p-STAT3 in the tMCAO brain (Fig. 6A). To further assess whether the neuroprotective effect of IL-13 is mediated by decreasing the activation of STAT3 (p-STAT3), we pretreated animals with STAT3 inhibitor V (stattic) prior to intranasal administration of IL-13 (Fig. 1A). As expected, the stattic injection effectively decreased p-STAT3 in the brain 3 days after tMCAO (Fig. 6A). Intranasal treatment of IL-13 after stattic injection did not further decrease p-STAT3 (Fig. 6A), possibly demonstrating that stattic and IL-13 reduced the activation of STAT3 in the ischemic brain by similar mechanisms.

IL-13 is an anti-inflammatory cytokine that has been reported to polarize microglia/macrophages to a beneficial anti-inflammatory phenotype [57–60]. Therefore, to evaluate whether IL-13 polarization of microglia/macrophage to an anti-inflammatory phenotype mediates its protective effects after ischemic stroke, we stained mouse brains 3 days after tMCAO with the microglia/macrophages marker Iba1, the anti-inflammatory phenotypic marker CD206, or the pro-inflammatory phenotypic marker CD16 (Fig. 6B). Indeed, although the four groups of mice showed a comparable number of Iba1 + microglia/macrophages after tMCAO (Fig. 6B), IL-13 or stattic treatment significantly reduced the number of pro-inflammatory microglia/macrophages (Fig. 6B, Pro) (CD16^+^CD206^−^Iba1^+^) and increased the number of anti-inflammatory microglia/macrophages (CD206^+^CD16^−^Iba1^+^) associated with inflammation resolution in the striatum (Fig. 6B, Resolve). Meanwhile, IL-13 or stattic treatment did not change the number of microglia/macrophages of the transitional (CD16^+^CD206^+^Iba1^+^) or resting (CD16^−^CD206^−^Iba1^+^) phenotypes in the striatum (Fig. 6B) after tMCAO. Importantly, IL-13 treatment after administration of stattic did not further reduce the number of pro-inflammatory microglia/macrophages or increase the number of anti-inflammatory microglia/macrophages (Fig. 6B).

### The transcription factor STAT3 plays a key role for the neuroprotective effects of IL-13 in ischemic stroke

**Fig. 7:**
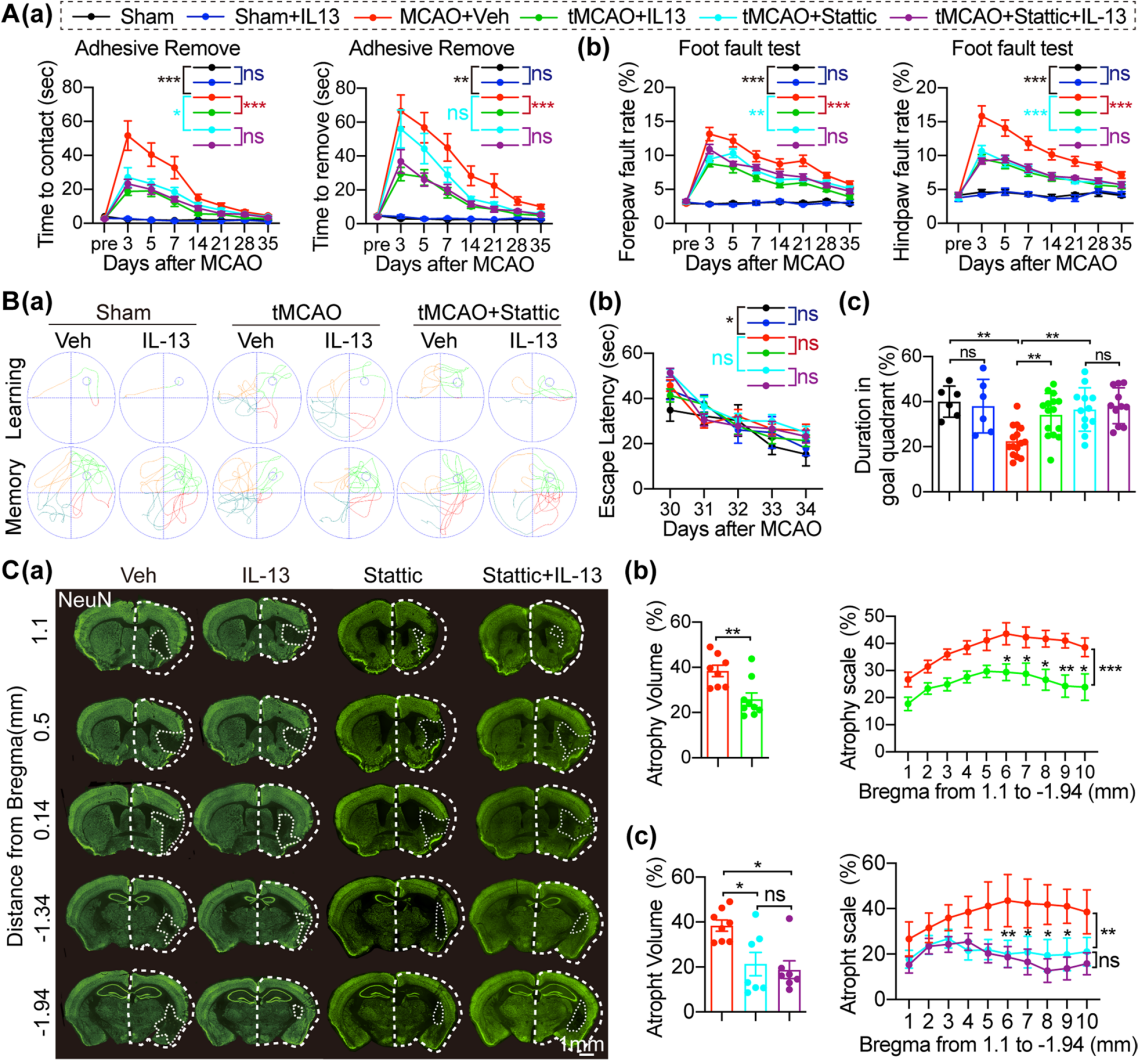
IL-13 mainly exerts neuroprotective effects by inhibiting the phosphorylation of STAT3. **A** Sensorimotor function was assessed by the adhesive removal test **Aa** and the foot fault test **Ab** up to 35d after tMCAO or sham operation. *n* = 6 mice for Sham + Veh and Sham + IL-13; *n* = 16 mice for tMCAO + Veh and tMCAO + IL-13; *n* = 12 mice for tMCAO + stattic; *n* = 11 mice for tMCAO + stattic + IL-13. **B** Long-term cognitive function was assessed by the MWM 30–35d after tMCAO or sham operation. **Ba** Representative swim paths. **Bb** Latency to find the hidden platform. **Bc** Time spent in the goal quadrant. *n* = 6 mice for Sham + Veh and Sham + IL-13; *n* = 15 mice for tMCAO + Veh and tMCAO + IL-13; *n* = 12 mice for tMCAO + stattic; *n* = 11 mice for tMCAO + stattic + IL-13. **C** Brain tissue loss 35d after tMCAO. **Ca** Representative images of NeuN (green) immunostaining. **Cb**, **c** Quantification of neuronal tissue loss. *n* = 7–8/groups. All data are presented as the mean ± SEM. *p ≤ 0.05, ** *p* ≤ 0.01, *** *p* ≤ 0.001. Two-way ANOVA followed by Bonferroni’s post hoc (**A**, **Bb**, **Cb**, **c**), one-way ANOVA followed by Bonferroni’s post hoc (**Bc**), Unpaired Student’s *t*-test (**Cb**), and Kruskal–Wallis test followed by Dunn’s post hoc (**Cc**)

We also assessed the effects of STAT3 inhibition with stattic on sensorimotor and cognitive function to explore whether it mimicked the neuroprotective effects of IL-13 in stroke mice (Fig. 1A). rCBF was measured in all stroke mice using laser speckle (Additional file 1: Fig. S7A) and laser Doppler flowmetry (Additional file 1: Fig. S7B). There was no difference in the percentage of blood flow decline in the four groups during tMCAO (Additional file 1: Fig. S7C). tMCAO mice treated with stattic showed a shorter time to contact and remove the tape in the adhesive removal test (Fig. 7Aa) and reduced forepaw and hindpaw foot fault rates in the foot fault test (Fig. 7Ab) compared to tMCAO mice treated with vehicle. Simultaneous administration of stattic and IL-13 did not further attenuate the neurological dysfunction compared to the tMCAO + stattic mice. In addition, in the MWM (Fig. 7B) the four surgery groups of mice showed comparable escape latency (Fig. 7Bb) and swimming velocity (Additional file 1: Fig. S7D). However, in the memory test, stattic-treated mice spent more time in the goal quadrant than the vehicle-treated group (Fig. 7Bc). Compared to the stattic-treated mice, IL-13 treatment after administration of stattic did not further attenuate the memory dysfunction caused by tMCAO (Fig. 7Bc). NeuN immunofluorescence staining was measured to assess neuron loss 35 days after tMCAO (Fig. 7C). Evaluation of cerebral tissue loss showed that IL-13 reduced the atrophy volume compared with vehicle treatment (Fig. 7Ca, b). Mice given stattic had a reduced atrophy volume compared to mice given vehicle (Fig. 7Ca, c), similar to what was observed with IL-13 treatment. Indeed, tMCAO + stattic mice and tMCAO + stattic + IL-13 mice showed comparable neuronal tissue loss (Fig. 7Cc).

### STAT3 is an important mediator of IL-13-induced white matter integrity maintenance and oligodendrogenesis after ischemic stroke

**Fig. 8:**
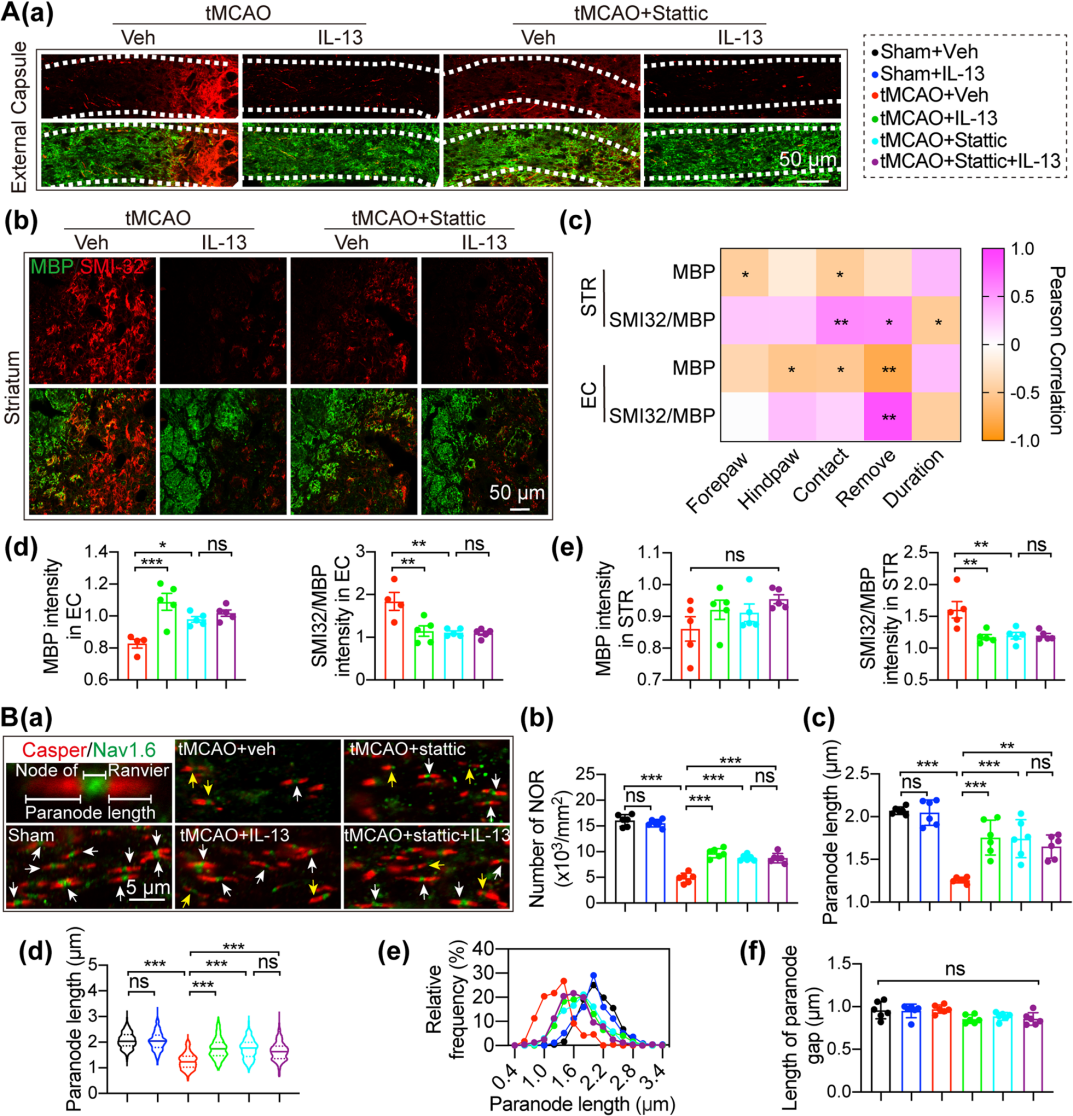
IL-13 promotes white matter integrity after stroke at least partially by inhibiting STAT3 activation. **A** Double-immunostaining for MBP and SMI32 35d after tMCAO. **Aa**, **b** Representative images of MBP (green) and SMI32 (red) immunostaining in the peri-infarct EC and STR. Scale bar: 50 µm. **Ac** The Pearson correlation between the behavior tests and histological analysis of myelin in the STR or EC. n = 4–5/group. **Ad**, **e** Quantification of the MBP fluorescence intensity and the ratio of SMI32 to MBP immunofluorescence intensity in ipsilateral EC and STR. n = 4–5/group. **B** Double-immunostaining for Caspr and Nav1.6 in the peri-infarct EC 35d after tMCAO. **Ba** Representative images of Caspr (red) and Nav1.6 (green) immunostaining. Scale bar: 5 µm. Yellow arrow: Typical NORs. White arrow: damaged NORs. **Bb**, **c** Quantification of the number of nodes of Ranvier (NOR) and the paranode length. **Bd** Violin plot of the paranode length and **Be** frequency histogram of the paranode length as a function of NORs. *n* = 252 NORs from six animals for sham + Vehicle; n = 251 NORs from six animals for sham + IL-13; *n* = 240 NORs from six animals for tMCAO + Vehicle; *n* = 246 NORs from six animals for tMCAO + IL-13; *n* = 241 NORs from six animals for tMCAO + Stattic; *n* = 239 NORs from six animals for tMCAO + stattic + IL-13. **Bf** Quantification of the length of paranode gap. *n* = 6/group. All data are presented as the mean ± SEM. *p ≤ 0.05, **p ≤ 0.01, ***p ≤ 0.001. One-way ANOVA followed by Bonferroni’s post hoc or Kruskal–Wallis test followed by Dunn’s post hoc (**Ad**, **e**). One-way ANOVA followed by Bonferroni’s post hoc (Bb-d), Kruskal–Wallis test followed by Dunn’s post hoc (**Bf**), and Pearson correlation (**Ac**)

White matter damage was also attenuated after inhibition of STAT3 with these treatments (Fig. 8). Stattic treatment improved MBP intensity in EC and decreased SMI32/MBP ratio in STR and EC (Fig. 8A) compared to vehicle treatment in mice 35 days after tMCAO, and no further improvement was observed when co-administered with IL-13 (Fig. 8A). The improvement in white matter integrity with IL-13/stattic treatment was associated with the sensorimotor improvement after tMCAO (Fig. 8Ac). Double-immunostaining of contactin-associated protein (Caspr) and sodium channel Nav1.6 were performed to evaluate the morphology of the nodes of Ranvier (NORs) 35 days after tMCAO (Fig. 8B). Typical NORs in sham EC are identified as the gap between the Caspr^+^ paranode and the Nav1.6^+^ nodes [61] (Fig. 8Ba). Stroke induced a decrease in the NOR number (Fig. 8Bb) and the paranode length (Fig. 8Bc-e) in the peri-infarct EC. Both IL-13 and stattic treatment attenuated this decrease in the NOR number (Fig. 8Bb) and paranode lengths (Fig. 8Bc-e) caused by tMCAO. Compared to stattic-treated mice, IL-13 treatment after administration of stattic did not further increase the number of NOR and paranode lengths (Fig. 8Bb-e). The lengths of the paranode gaps were comparable between post-ischemic and non-injured EC (Fig. 8Bf).

**Fig. 9:**
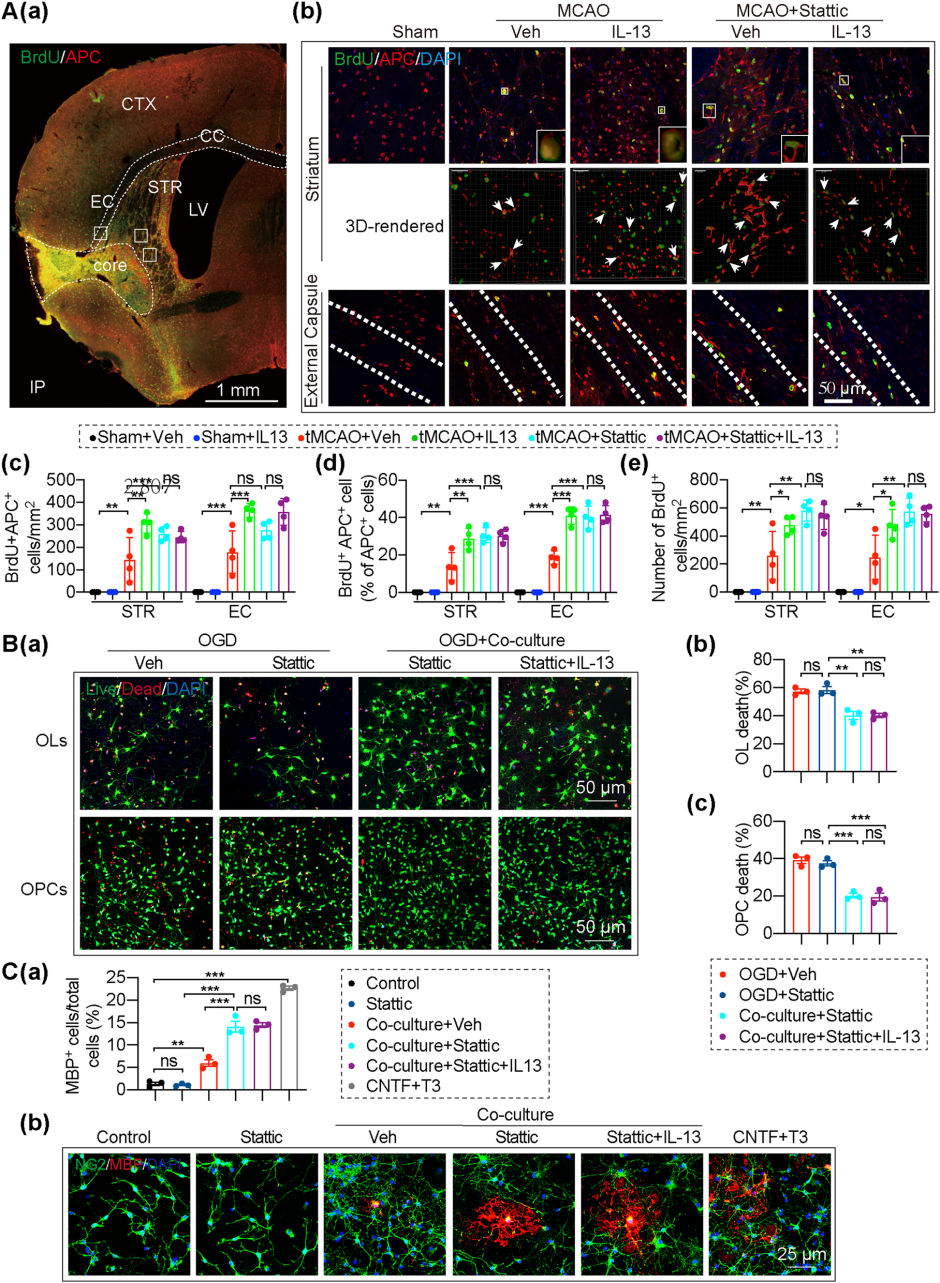
IL-13 promotes oligodendrogenesis after stroke at least partially by inhibiting STAT3 activation. **A** Double-immunostaining for BrdU and APC 35d after tMCAO. **Aa** illustrates where images in **b** were taken from. **Ab** Representative images of BrdU (green) and APC (red) immunostaining in the peri-infarct STR and EC. Scale bar: 50 µm. White squares illustrate the location of 3D-rendered images magnified. **Ac**–**e** Quantification of the number of BrdU^+^ cells and BrdU^+^APC^+^ cells in the peri-infarct STR and EC. *n* = 4/group. **B** OL/OPC survival after OGD/R was quantified by live/dead staining. **Ba** Representative images of live (green) and dead (red) staining of cultured oligodendrocytes or OPCs. Scale bar: 50 μm. **Bb**, **c** Percentages of dead OLs and OPCs were quantified. Data are from 3 independent experiments. **C** NG2 and MBP immunostaining was performed to evaluate OPC differentiation. **Ca** Percentages of MBP^+^ OLs were quantified. Data are from 3 independent experiments. **Cb** Representative images of NG2 (green), MBP (red) and DAPI (blue) staining of cultured oligodendrocytes or OPCs. Scale bar: 25 μm. All data are presented as the mean ± SEM. *p ≤ 0.05, **p ≤ 0.01, ***p ≤ 0.001. One-way ANOVA followed by Bonferroni’s post hoc, or Kruskal–Wallis test followed by Dunn’s post hoc

Successful differentiation of OPCs into myelinating oligodendrocytes is important for remyelination [34]. Thus, we investigated if IL-13 treatment promotes oligodendrogenesis by inhibiting STAT3 activation. Newly generated mature OLs were labeled with antibodies against BrdU (marker for newly generated cells) and APC (marker for mature OLs) 35 days after tMCAO (Fig. 9A). Stroke induced spontaneous oligodendrogenesis in the peri-lesional STR and EC. Compared to vehicle-treated mice, a significantly higher ratio of BrdU^+^APC^+^ newly generated oligodendrocytes was observed in both IL-13 and stattic-treated mice 35 d after MCAO (Fig. 9Ac, d). The total numbers of BrdU^+^ cells were also significantly increased in both IL-13 and stattic-treated mice compared to vehicle-treated mice (Fig. 9Ae). Compared to stattic-treated mice, IL-13 treatment after administration of stattic did not further increase the number of BrdU^+^APC^+^ OLs or BrdU^+^ cells (Fig. 9Ac–d).

### STAT3 activation in microglia is essential for IL-13-induced OPC/OL protection and OPC differentiation

To further assess whether the oligoprotective effects of IL-13 is mediated by decreasing the activation of STAT3 in microglia, we treated mice primary microglia with stattic (2.5 μM/mL) and co-culture with the OPCs or OLs that had been subjected to 1 h OGD for 24 h (Fig. 9B). As expected, direct stattic treatment on primary oligodendrocytes or OPC cultures failed to protect them against OGD/R (Fig. 9B). Interestedly, the co-culture of stattic-treated microglia significantly reduced OL and OPC death after OGD/R (Fig. 9B). Compared to stattic-treated microglia and OL/OPC co-cultures, IL-13 and stattic simultaneous treatment group did not further reduce OL and OPC death after OGD/R (Fig. 9B). Next, we investigated the impact of stattic on OPC differentiation (Fig. 9C). Interestedly, stattic (2.5 μM/mL) had no direct effects on OPC differentiation, but stattic-treated primary microglia and OPC co-cultures showed significantly increased MBP^+^ mature OLs compared with co-cultured primary microglia and OPC without stattic treatment. Importantly, in the experiment co-cultured with microglia, IL-13 and stattic simultaneous treatment group did not further encourage OPC differentiation, compared with stattic treatment group (Fig. 9C). Collectively, these data demonstrate that IL-13 promotes OPC differentiation and OPC/OL survival after OGD/R by inhibiting the activation of STAT3 in microglia.

## Discussion

Previous studies examining the effect of IL-13 against stroke have focused on its effects on microglia/macrophage activation and short-term functional recovery [47, 48]. The long-term neuroprotective effects of IL-13 after stroke remain largely unexplored. Therefore, in the present study, we investigated the effect of IL-13 on ischemic stroke in a mouse tMCAO model up to 35 days post-stroke. Intranasal administration of IL-13 after tMCAO attenuated sensorimotor and cognitive deficits, which correlated with a decrease in white matter damage, lending further support that loss of white matter integrity is strongly linked to poor stroke outcomes in ischemic stroke patients [33]. These data corroborate results observed in previous studies that IL-13 can improve neurological deficits and brain damage after stroke (up to 14 days) [47, 48] and shows that this improvement may persists for longer times post-stroke (up to 35 days post-stroke). Our in vitro results further indicate that the neuroprotection induced by IL-13 treatment was not due to direct effects on neurons and OPCs/OLs, but in a microglia-dependent manner. These findings are limited to the simulation of ischemic stroke through OGD/R of neurons and OPCs/OLs in vitro. IL-13 treatment decreased the expression of multiple pro-inflammatory factors, including TNF-α, CD16, and CD86. The reason why IL-6 expression was not detected is possibly because the time point chosen is too late, since IL-6 marks the onset of ischemic stroke. Notably, we have shown in the current study that this IL-13-induced anti-inflammatory shift in microglia/macrophage, matter integrity maintenance and oligodendrogenesis were mediated, at least partially, by inhibiting STAT3 activation in the cerebrum after tMCAO, a previously undefined mechanism of IL-13 in stroke. Our in vitro results further indicate that the activation of STAT3 in microglia is essential for IL-13-induced OPC/OL protection and OPC differentiation. Whether IL13 targets other molecules warrants further investigation. Our research reveals for the first time that IL-13 improves long-term neurological deficits and white matter damage caused by stroke, which provides pre-clinical rationale for the application of IL-13 in future investigations of stroke.

The mechanism(s) by which IL-13-induced alterations in STAT3 signaling modifies ischemic stroke outcomes remains to be clarified. In the adult nervous system, the JAK2-STAT3 pathway is mostly dormant but can be activated rapidly in vitro and in vivo by ischemic stress [62–66]. However, there are conflicting data as to whether activation of this pathway leads to improved neurological recovery in stroke. A recent study on non-erythropoietic mutant erythropoietin (MEPO) showed that increased STAT3 phosphorylation was involved in the neuronal protection provided by MEPO in stroke mice using the specific inhibitor AG490 to block JAK2/STAT3 activation [66]. In contrast, accumulating evidence also shows that activation of the JAK2/STAT3 signaling pathway can increase the expression of the high mobility group box 1 protein or HMGB1, leading to aggravation of post-ischemic inflammatory responses [67–69] and that reducing STAT3 activation improves functional performance and decreased neuronal damage after stroke [16, 65]. STAT3 activation has been shown to mediate pro-inflammatory responses in microglia in response to ischemic stroke [17], whereas inhibition of the JAK2-STAT3 pathway has been shown to selectively promote microglia polarization to a beneficial phenotype [70]. These latter studies are in line with our data, as we observed a phenotypic shift in favor of microglia/macrophage involved in inflammation resolution, which support the beneficial effect of reducing p-STAT3 after stroke. However, microglia/macrophages specific inhibition approach to target STAT3 in vivo has not yet been explored. Systemic administration of stattic does not allow excluding possible effects of this compound in the periphery, which is a limitation of the study. Nonetheless, the clinical application prospect of stattic in the after stroke warrants further investigation.

In addition to STAT3, STAT6 also acts downstream of the heterodimer of the IL-4Rα and IL-13Ralpha1 receptors, which enter the nucleus after activation to initiate transcription of anti-inflammatory genes in microglia/macrophages [71]. Whether IL-13 regulates STAT6 signaling in response to ischemic stroke remains to be determined. Signaling through IL-4 receptors on macrophages induces activation of STAT6, and the magnitude of STAT6 activation induced by IL-4 in macrophages is generally greater than that induced by IL-13 [72]. IL-4 was able to stimulate phosphorylation of STAT6 at significantly lower doses than IL-13 in the human epithelial carcinoma cell line A549 [73], suggesting that STAT6 may not be the main downstream molecular target of IL-13. Thus in the current study we focused on STAT3 as a potential downstream target of IL-13 signaling.

IL-13 signaling is initiated by binding to the IL-13 receptor, a heterodimer of the alpha chain of the IL-4 receptor, which also binds IL-4, and IL-13-Ralpha 1 [13]. IL-4 is secreted by ischemic neurons as an endogenous defense mechanism [74], and we have previously reported that IL-4 receptors are expressed in oligodendrocyte lineage cells [34] and their stimulation with IL-4 promoted oligodendrogenesis after traumatic brain injury (TBI) [75]. IL-13 is mainly expressed in microglia in the brain [76] and LPS induces IL-13 expression exclusively in activated microglia [8]. In the current study, we demonstrated that IL-13Rα1 was mainly expressed on microglia/macrophages and neurons under both physiological and under stroke conditions, with hardly any expression on oligodendrocyte. This suggests that although IL-4 and IL-13 share the same receptor, they may function through completely different cells after a stroke.

The IL-13Rα2 receptor also binds IL-13, with even higher affinity than IL-13Rα1 [77]. Although some studies have shown evidence of IL-13Rα2-mediated signal transduction [45, 78], IL-13Rα2 is believed to act as a decoy receptor, and through this role, overexpression of IL-13Rα2 could in fact diminish IL-13 signaling. Because of this reputed role, along with its limited expression in the stroke brain and the lack of clarity regarding its biological role [79], we did not examine the role of IL-13Rα2 in IL-13 signaling in the present study. Nonetheless, the role of IL-13Rα2 in IL-13 signaling warrants further investigation.

In addition to the central location of IL-13 receptors in the brain, IL-13 also acts through IL-13 receptors that are widely expressed on peripheral immune cells [12]. Nasal drug delivery is a form of peripheral administration and IL-13 is a pleiotropic type 2 cytokine that contributes to eosinophil chemotaxis in eosinophilic esophagitis (EOE) and atopic dermatitis (AD) [37, 38]. IL-13 induces infiltration of eosinophils, neutrophils, lymphocytes, and IL-17^+^CD4^+^ T cells into the bronchoalveolar compartment of the lung in allergic airway inflammation [39, 80, 81]. We hereby proved that intranasal IL-13 treatment neither changed the infiltration of peripheral immune cells, which enters the brain to participate in central immunity after a stroke, nor did it change the immune cell population in blood, spleen and lung. The lack of any change in peripheral immune responses after intranasal IL-13 application is consistent with our previous TBI study [18]. These results indicate that the intranasal IL-13 dose (60 μg/kg) used in this study had little effect on peripheral immune cells, which may be because the dose used was much lower than the dose of IL-13 intratracheally administered (10 μg/mice) to induce airway hyperreactivity in the allergic lung [82]. Thus, intranasal delivery of IL-13 may be an attractive delivery method in which to circumvent off target effects. Ample proofs have shown that drugs can be directly delivered to the CNS via the nasal route, but the brain direct transport pathways are extremely complicated. *Hongbing Wu *et al*.* summarized three major recognized drug pathways into the brain upon absorption via nasal mucosa: (1) olfactory nerve pathway: absorption is slow, with a duration from 1 – 2 to 24 h, depending on the process of pinocytosis, internalization or simple diffusion, and then the axoplasm flow of olfactory neurons to the olfactory bulbs and further to the rhinencephalon; (2) olfactory mucosa epithelium pathway: this acts within minutes, the substances absorbed into the lamina propria enter the CNS through the gaps surrounding the olfactory nerve tract; (3) blood circulation pathway: substances are absorbed through blood capillaries or respiratory mucosa, or enter the blood circulation via lamina propria of the olfactory region, then pass the BBB to CSF or brain tissues with different durations. Therefore, drugs may enter the CNS via one or several transport mechanisms over different periods of time [41]. In view of this, the absorption efficiency and content of IL-13 in all parts of the brain after intranasal IL-13 treatment were not explored in this research. The reported concentrations do not reflect the exposure of target cells to IL-13.

In addition to microglia/macrophages, infiltrating neutrophils and brain-intrinsic astrocytes also display heterogeneity in activation. After stroke, the population of infiltrated neutrophils in the brain is heterogeneous, including a population of alternative neutrophils (N2) that express M2 phenotype markers, which was associated with neuroprotection and resolution of inflammation after experimental stroke, as opposed to a pro-inflammatory neutrophil subset found in tumors previously that was named N1 [83]. Astrocytes are another type of glial cells that actively participate in regulation of neuroinflammation [84]. Neuroinflammation and ischemia induced two different types of reactive astrocytes, corresponding to “A1” neurotoxic and “A2” neuroprotective, respectively, which are similar to the “M1-like” and “M2-like” of microglia [85].

The current understanding of the mechanisms of ischemic brain injury includes an appreciation of multicellular interactions within the neurovascular unit (NVU), which may determine the evolution of blood–brain barrier (BBB) damage, neuronal cell death, glial reaction, and immune cell infiltration [86]. In the current study we did not conduct research on whether IL-13 treatment changes BBB damage, nerve regeneration, etc., which needs to be confirmed by further research.

## Conclusions

In conclusion, we reported a previously undefined molecular target of IL-13 in the CNS, namely that IL-13 played a neuroprotective role at least partially by inhibiting the activation of STAT3, which has been shown to mediate pro-inflammatory responses in microglia in response to ischemic stroke. Inhibition of p-STAT3 is involved in the microglia/macrophages polarization, white matter repair and long-term neuronal protection provided by IL-13 after ischemic stroke. Accordingly, IL-13 is an attractive, novel therapeutic target in ischemic stroke. Our investigation thus provides a pre-clinical rationale for the application of IL-13 as a potential treatment for stroke.

## Supplementary Information

**Additional file 1.** Figure S1–S7.

## Data Availability

The datasets used and analyzed during the current study are available from the corresponding author on reasonable request.

## Abbreviations

CNS: Central nervous system
LPS: Lipopolysaccharide
CC: CorPus callosum
CTX: Cortex
EC: External capsule
STR: Striatum
IL-13: Interleukin-13
IL-13Rα1: IL-13 receptor alpha1
IL-13Rα2: IL-13 receptor alpha2
MBP: Myelin basic protein
MG: Microglia
MΦ: Macrophages
MWM: Morris water maze test
SMI32: Non-phosphorylate neurofilament heavy polypeptide
STAT3: Signal transducer and activator of transcription 3
p-STAT: Phosphorylated-STAT3
TEM: Transmission electron microscopy
JAK: Janus kinase
tMCAO: Transient middle cerebral artery occlusion
TBI: Traumatic brain injury
rCBF: Regional cerebral blood flow
OPCs: Oligodendrocyte precursor cells
Ols: Oligodendrocytes
