# Supplementary material for: Interleukin 13 promotes long-term recovery after ischemic stroke by inhibiting the activation of STAT3
Source: J Neuroinflammation. 2022 May 16;19:112. doi: 10.1186/s12974-022-02471-5 (PMC9109418; doi:10.1186/s12974-022-02471-5)
Supplement: Supplementary file 1 — Additional file 1. Figure S1–S7. [file 12974_2022_2471_MOESM1_ESM.docx]

**Additional file 1**

**Interleukin 13 Promotes long-term recovery after Ischemic Stroke by Inhibiting the Activation of STAT3**

Di Chen, Jiaying Li, Yichen Huang, Pengju Wei, Wanying Miao, Yaomei Yang, Yanqin Gao

State Key Laboratory of Medical Neurobiology, MOE Frontiers Center for Brain Science, and Institutes of Brain Science, Fudan University, Shanghai 200032, China

Address correspondence

Dr. Yanqin Gao,

State Key Laboratory of Medical Neurobiology, MOE Frontiers Center for Brain Science, and Institutes of Brain Science, Fudan University,

138 Yixueyuan Road, Shanghai 200032.

Telephone/Fax :8621-54237778.

E-mail address: yqgao@shmu.edu.cn

Additional file 1: Figures 1-7

**Supplemental Figures**


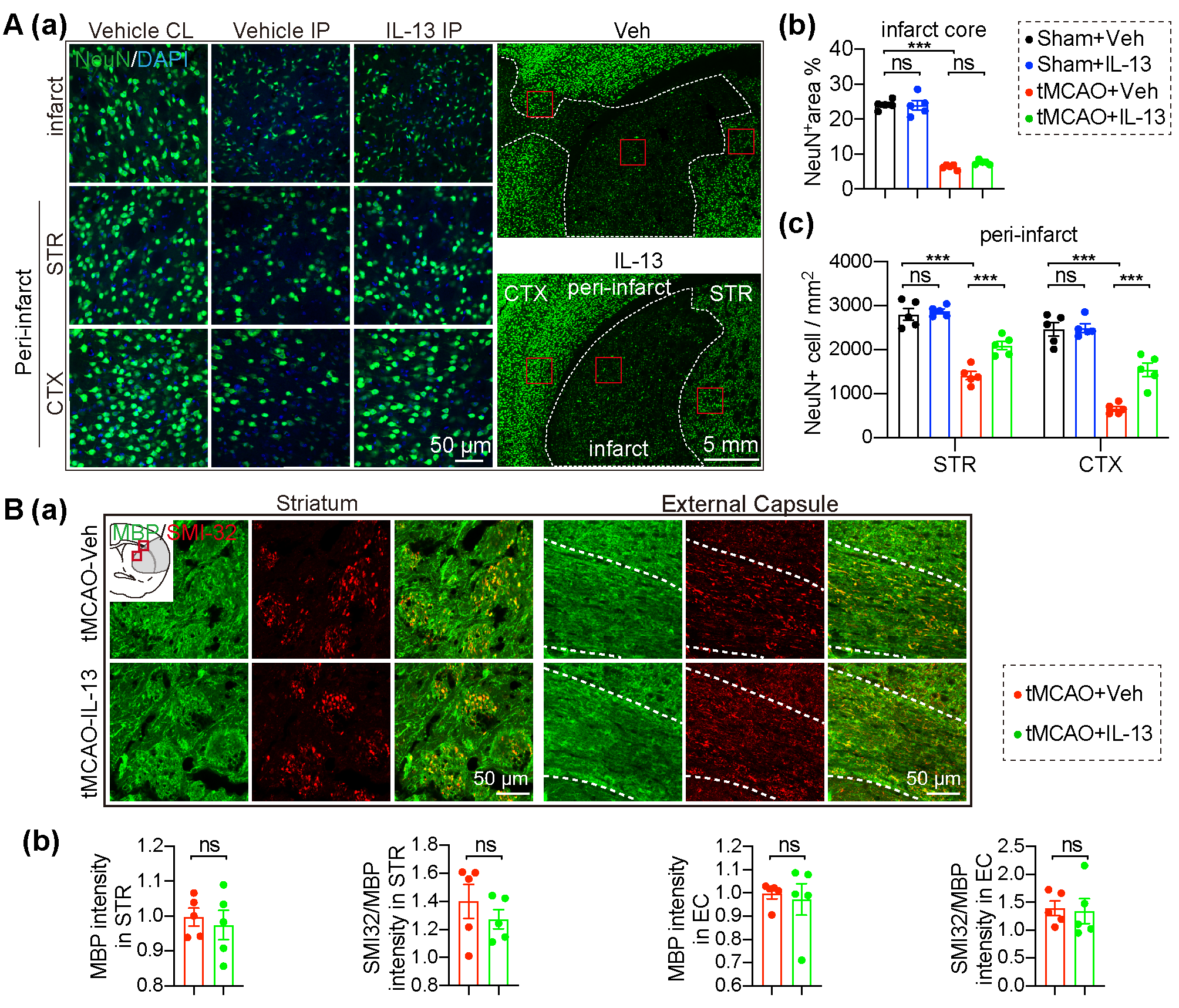


**Figure S1. IL-13 improves neuron loss but did not reduce the demyelination damage caused by tMCAO during the acute phase of stroke. (A)** Neuron loss was quantified on NeuN (green) immunostained coronal sections. **(Aa)** Representative images of NeuN (green) and DAPI (blue) immunostaining in coronal brain sections 3d after tMCAO. Scale bar: 50 µm. Dashed line: infarct border. Red rectangles: illustrates where images in the left panels were taken from. **(Ab-c)** Quantification of the density of neurons based on NeuN immunostained brain slices. n = 5/groups. **(Ba)** Representative images of MBP (green) and SMI32 (red) immunostaining in the STR and EC. Scalebar: 50 µm. **(Bb)** Quantification of the MBP fluorescence intensity and the ratio of SMI32 to MBP immunofluorescence intensity in ipsilateral STR and EC. n = 5/group. All data are presented as the mean ± SEM. One-way ANOVA followed by Bonferroni’s post hoc (A),Mann-Whitney U test or Unpaired Student’s t-test (Bb).

**
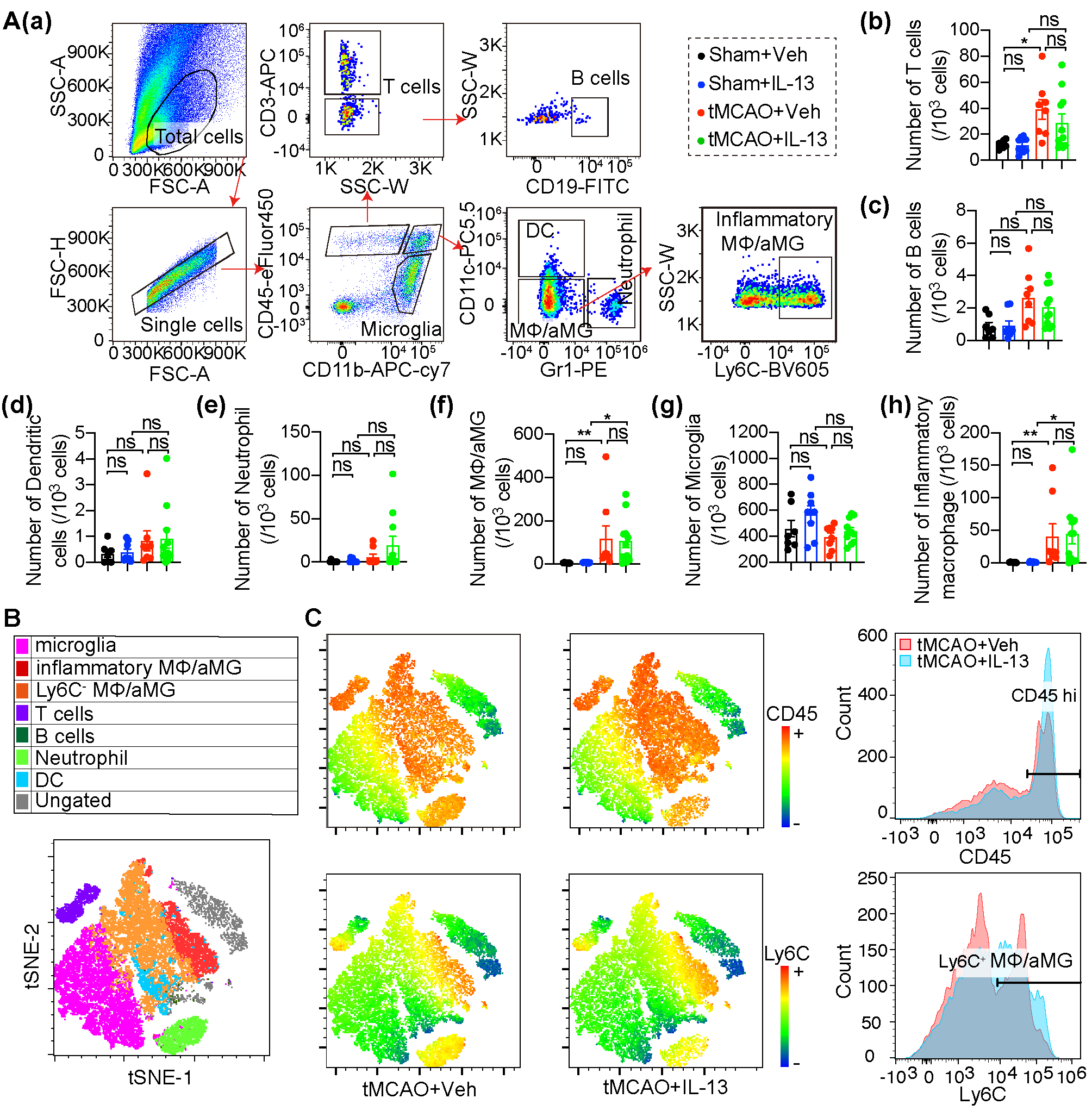
**

**Figure. S2. Intranasal IL-13 treatment does not alter the adaptive immune cell population in brain after stroke. (A-C)** The infiltration of peripheral immune cells into the brain was assessed by flow cytometry. **(Aa)** Gating strategy for the lymphocyte populations in the brain. **(Ab-h)** Quantification of lymphocyte populations in the brain by flow cytometry，including T lymphocytes **(Ab)**, B lymphocytes **(Ac)**, dendritic cells **(Ad)**, neutrophils **(Ae)**, macrophages **(Af)**, microglia **(Ag)** and inflammatory macrophages **(Ah)**. n=7-11/group. **(B-C)** A tSNE ( t-distributed Stochastic Neighbor Embedding) projection of 2 tMCAO+Veh mice and 2 tMCAO+IL13 mice. **(B)** Representative tSNE plots of 60000 single cells pooled from the tMCAO+ Vehicle and tMCAO+IL13 groups. Each dot represents one cell. Cells were color coded by their type. **(C)** Representative tSNE plots of 30000 single cells pooled from the tMCAO+Vehicle and 30000 single cells pooled from the tMCAO+IL13 groups. Each dot represents one cell. Cells were color coded by their expression level of CD45 or Ly6C. All data are presented as the mean ± SEM. *P ≤ 0.05, **P ≤ 0.01, ***P ≤ 0.001. One-way ANOVA followed by Bonferroni’s post hoc, or Kruskal-Wallis test followed by Dunn’s post hoc.


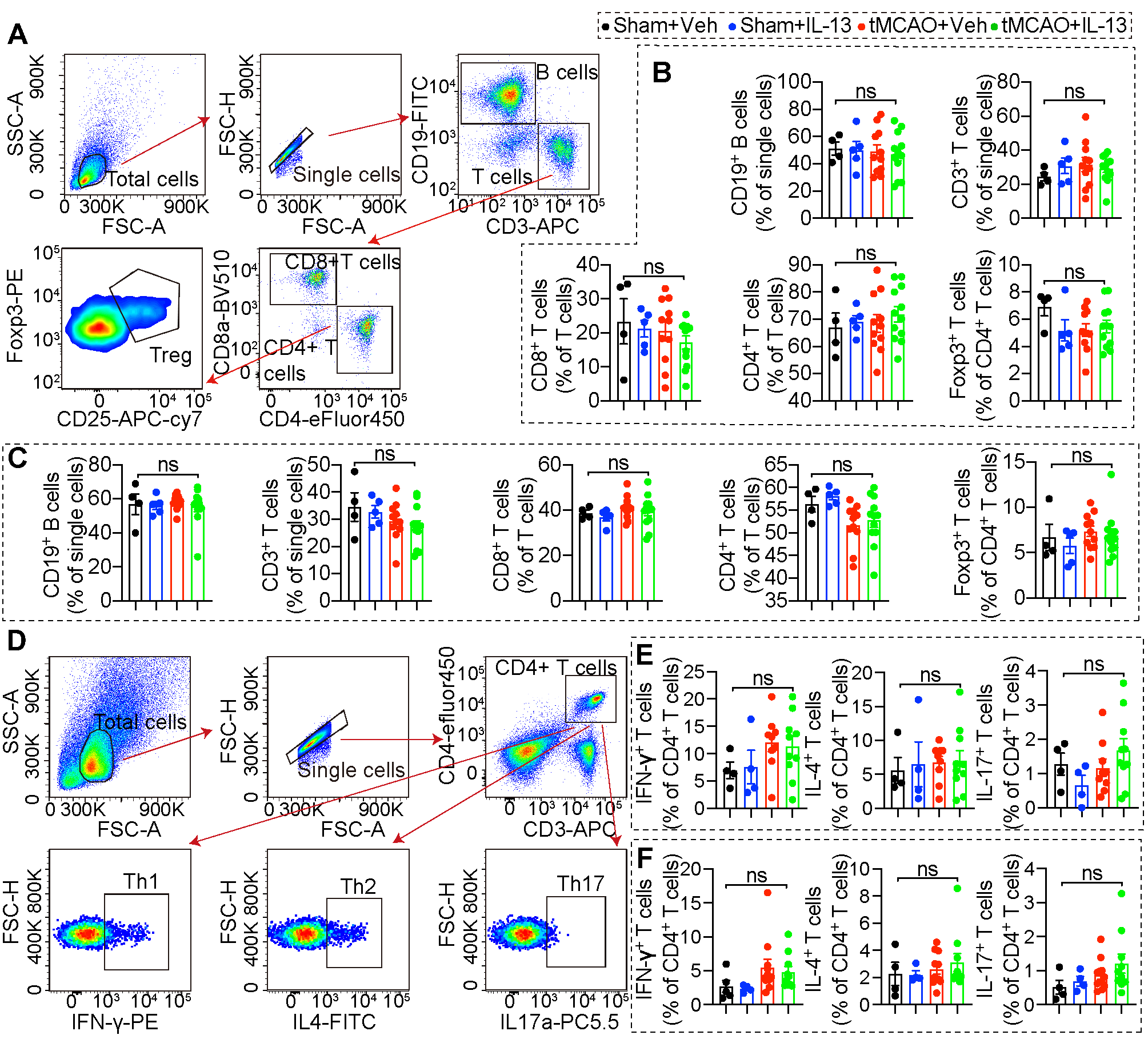


**Figure S3. The lymphocyte subsets in the blood and spleen were assessed by flow cytometry three days after tMCAO.** **(A)** Gating strategy for the lymphocyte populations in the blood and spleen. **(B)** Quantification of lymphocyte populations in the blood by flow cytometry. **(C)** Quantification of lymphocyte populations in the spleen by flow cytometry. n = 4 mice for Sham + Vehicle; n = 5 mice for Sham + IL-13; n = 11 mice for tMCAO + Vehicle and n = 12 mice for tMCAO + IL-13. **(D)** Gating strategy for the subpopulations of CD4^+^ T cells in the blood and spleen. **(E)** Quantification of subpopulations of CD4^+^ T cells in the blood by flow cytometry. n = 4 mice for Sham + Vehicle and Sham + IL-13; n = 9 mice for tMCAO + Vehicle and n = 10 mice for tMCAO + IL-13. **(F)** Quantification of subpopulations of CD4^+^ T cells in the spleen by flow cytometry. n = 4 mice for Sham + Vehicle and Sham + IL-13; n = 11 mice for tMCAO + Vehicle and n = 10 mice for tMCAO + IL-13. All data are presented as the mean ± SEM. One-way ANOVA followed by Bonferroni’s post hoc or Kruskal-Wallis test followed by Dunn’s post hoc.


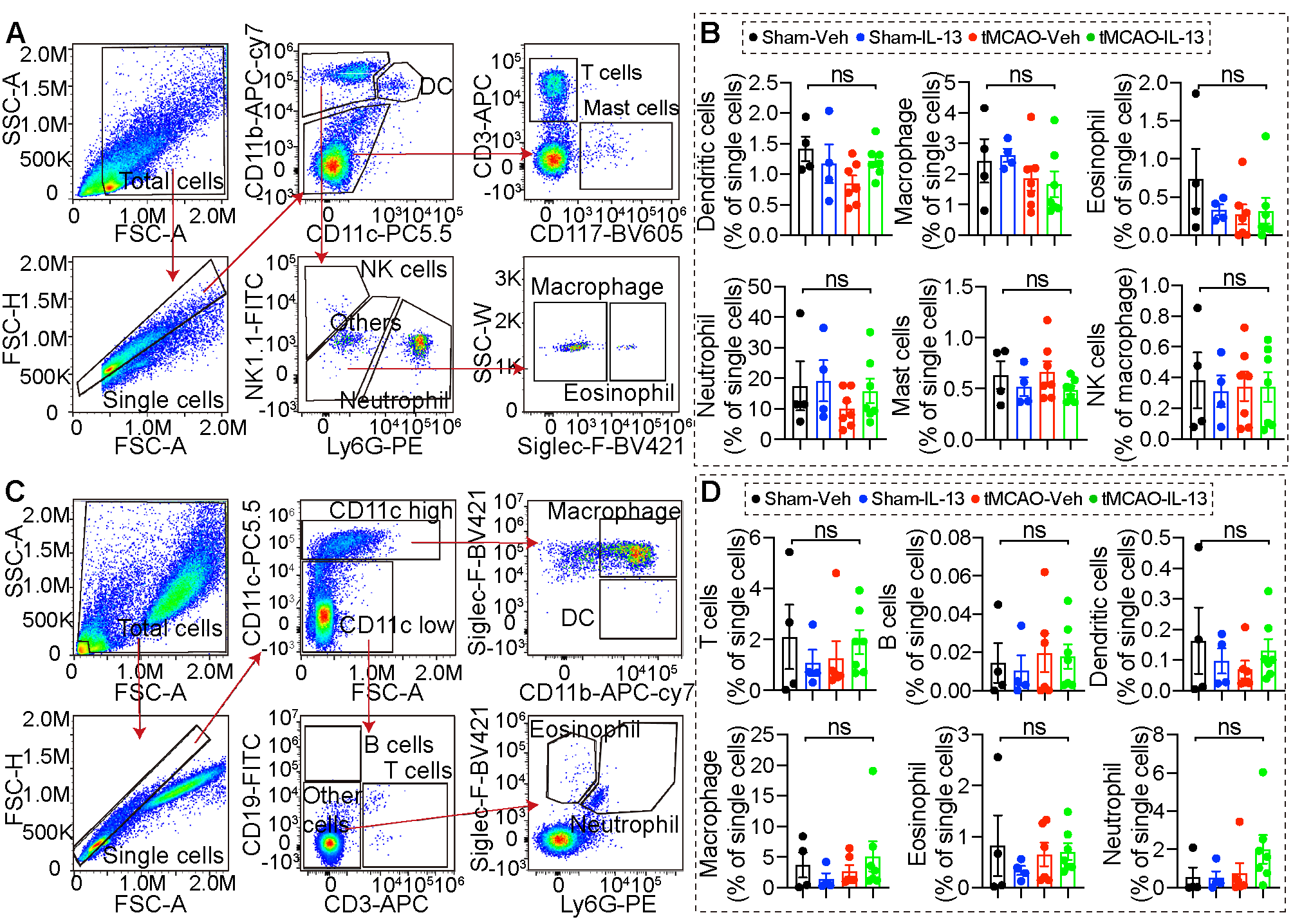


**Figure S4. The function-related immune cells in the blood and lung were assessed by flow cytometry three days after tMCAO. (A)** Gating strategy for the function-related immune cells in the peripheral blood. **(B)** Quantification of lymphocyte populations in the blood by flow cytometry. n = 4 mice for Sham + Vehicle and Sham + IL-13; n = 7 mice for tMCAO + Vehicle and tMCAO + IL-13. **(C)** Gating strategy for function-related immune cells in the lung. **(D)** Quantification of function-related immune cells in the lung lavage fluid by flow cytometry. n = 4 mice for Sham + Vehicle and Sham + IL-13; n = 6 mice for tMCAO + Vehicle and n = 7 mice for tMCAO + IL-13. All data are presented as the mean ± SEM. One-way ANOVA followed by Bonferroni’s post hoc or Kruskal-Wallis test followed by Dunn’s post hoc.


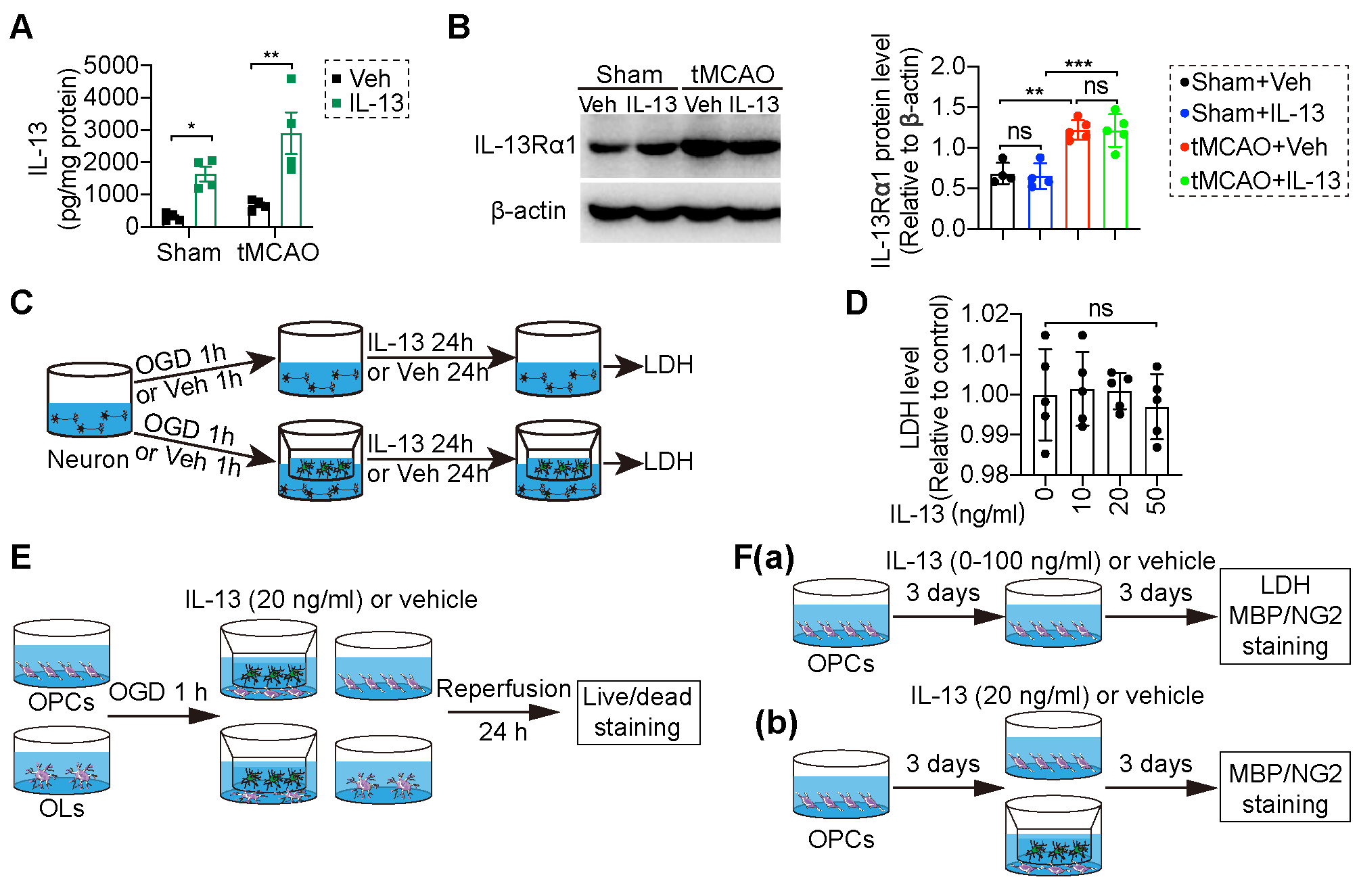


**Figure S5. The expression level of IL-13Rα1 increased significantly after tMCAO and the design of in vitro experiments. (A)** Brain IL-13 protein levels were detected by ELISA three hours after IL-13 or vehicle administration at 3d after tMCAO or sham operation. n = 4/group. **(B)** Brain IL-13Rα1 expression was measured using Western blots 3d after tMCAO or sham operation. Representative Western blots of IL-13Rα1 and β-actin and quantification of IL-13Rα1 expression in Western blots. n = 4-5/group. **(C)** Experimental design of *In vitro* experiments of neuron. **(D)** LDH was measured to evaluate the cytotoxicity of different concentrations of IL-13 (10 -50 ng/ml) on mouse primary microglia. n = 5/group. **(B)** Schematic illustration of the Figure 6A experimental design. **(C)** Schematic illustration of the Figure 6D-F experimental design. All data are presented as the mean ± SEM. *P ≤ 0.05, **P ≤ 0.01, ***P ≤ 0.001. Two-way ANOVA followed by Bonferroni’s post hoc (A), One-way ANOVA followed by Bonferroni’s post hoc (B, D).


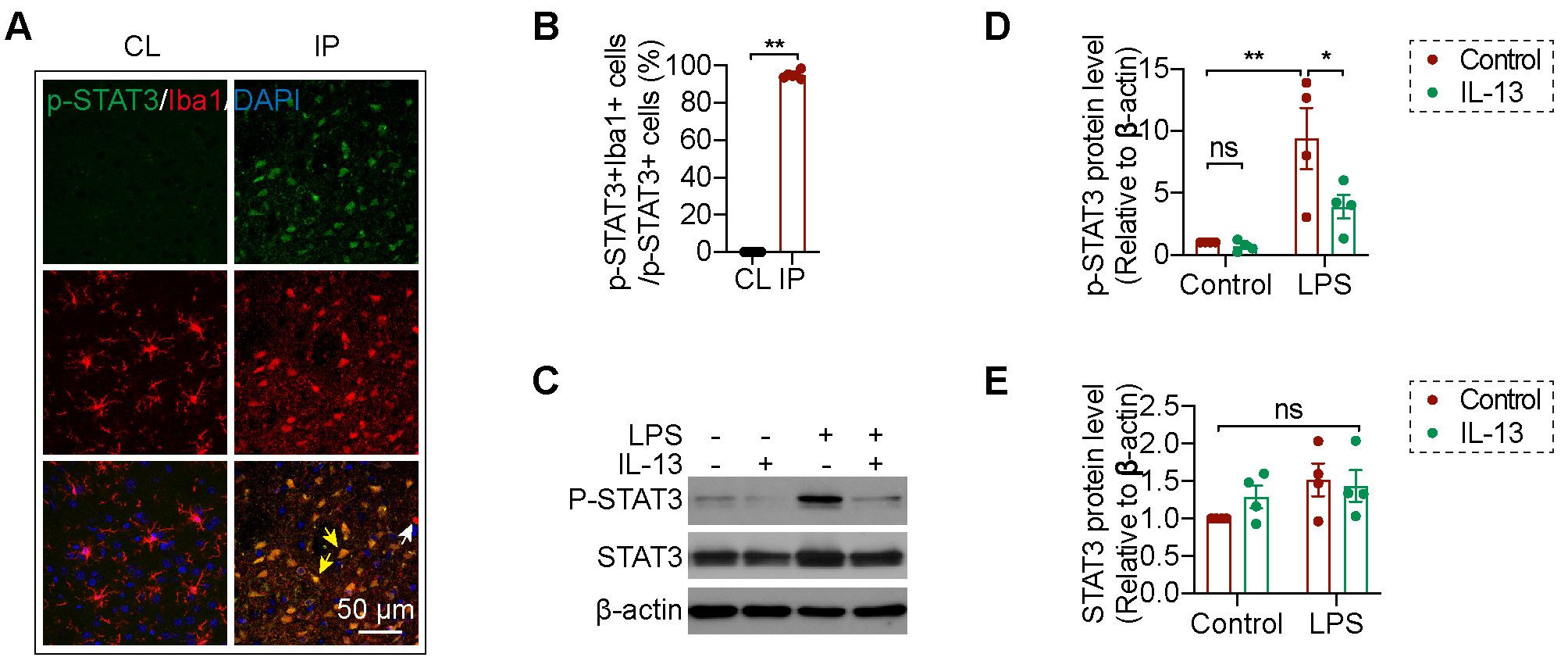


**Figure S6. IL-13 inhibits STAT3 activation in microglia. (A)** Double immunostaining for p-STAT3 and Iba1 3d after tMCAO. **(A)** Representative images of p-STAT3 (green), Iba1 (red) and DAPI (blue) immunostaining in the infarct striatum. Yellow arrow: p-STAT3^+^Iba1^+^. White arrow: p-STAT3^-^Iba1^+^. Scalebar: 50 µm. **(B)** Quantification of the p-STAT3^+^Iba1^+^ cells in the infarct striatum. n = 6/group. **(C-E)** p-STAT3 and STAT3 expression in primary cultured microglia treated with LPS (100 ng/ml) and/or IL-13 (20 ng/ml) was measured using Western blots at 24h after treatment. **(C)** Representative Western blots of p-STAT3, STAT3, and β-actin in primary microglia. **(D-E)** Quantification of p-STAT3 and STAT3 expression in Western blots. n = 4/group. All data are presented as the mean ± SEM. *P ≤ 0.05, **P ≤ 0.01. Mann-Whitney U test (B), Two-way ANOVA followed by Bonferroni’s post hoc (D-E).


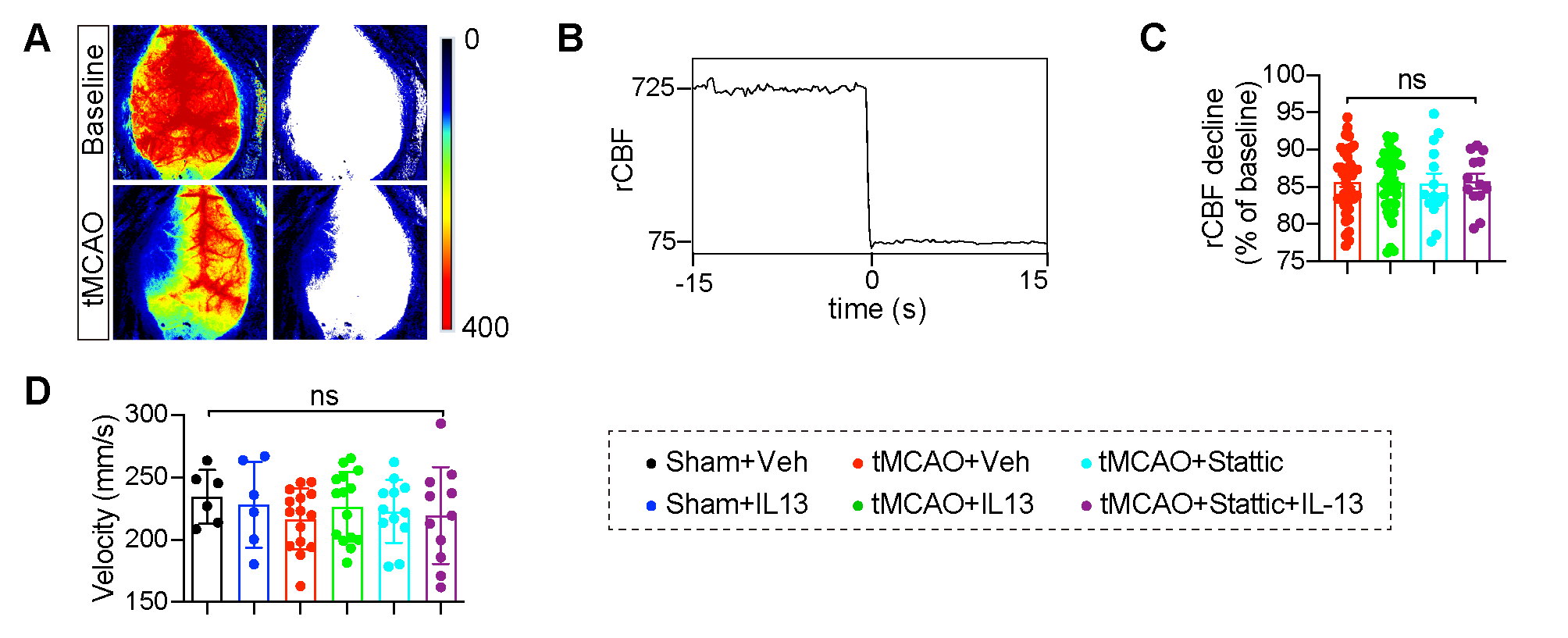


**Figure S7. The rCBF during tMCAO surgical operation and the velocity in the Morris water maze. (A)** rCBF 10 minutes before and 10 minutes after tMCAO operation detected by laser speckle. The image on the right shows the area where rCBF has dropped by more than 70%. **(B-C)** Regional cerebral blood flow was measured by laser doppler flowmetry. **(D)** Velocity in the Morris water maze. All data are presented as the mean ± SEM. *P ≤ 0.05, **P ≤ 0.01. One-way ANOVA followed by Bonferroni’s post hoc.
